# Incorporating Phylogeography and SDM Projection to Inform Conservation Efforts of the Foundation Seaweed *Gloiopeltis furcata* sensu lato in the North Pacific Under Climate Change

**DOI:** 10.1111/eva.70323

**Published:** 2026-09-23

**Authors:** Qi‐Qi Wang, Mi Yeon Yang, Myung Sook Kim, Juan Diego Gaitan‐Espitia, Xuan Vy Nguyen, Jun‐Mei Qu, Zhixin Zhang, Zi‐Min Hu

**Affiliations:** ^1^ School of Ocean Yantai University Yantai China; ^2^ Department of Biology and Research Institute for Basic Sciences Jeju National University Jeju South Korea; ^3^ Department of Biological Sciences Kangwon National University Gangneung South Korea; ^4^ The Swire Institute of Marine Science, School of Biological Sciences The University of Hong Kong Hong Kong China; ^5^ Department of Marine Botany Institute of Oceanography, Vietnam Academy of Sciences and Technology Nha Trang Ward Khanh Hoa Province Viet Nam; ^6^ State Key Laboratory of Tropical Oceanography South China Sea Institute of Oceanology, Chinese Academy of Sciences Guangzhou China; ^7^ Global Ocean and Climate Research Center South China Sea Institute of Oceanology, Chinese Academy of Sciences Guangzhou China

**Keywords:** biodiversity conservation, distance to shore, genetic lineages, habitat suitability, mitochondrial *cox*1, range shifts, species distribution modelling

## Abstract

Understanding the impact of past climate events on population genetic structuring and range shifts may facilitate predictions of how species will respond to future climate change. Such integrative effort, however, remain rare for the North Pacific benthic ecosystem. Here, we obtained mtDNA *cox*1 and cpDNA *rbc*L sequences of 85 populations of the foundation macroalga *Gloiopeltis furcata* sensu lato (s.l.) in the North Pacific to estimate its lineage structuring and phylogeographic connectivity. We found that *G. furcata* s.l. harbored high and cryptic lineage diversity, and its present‐day biogeographic patterns are mostly attributed to extensive vicariance and ocean‐currents mediated dispersal events. In particular, the southwestern Japanese Archipelago and southern Korea potentially served as the center of ancestral persistence during the maximum ice sheet expansion, characterized by concurrent demographic contractions of lineages. We further applied projection‐based species distribution models (SDMs) and detected consistent habitat suitability contraction in low latitude regions (20°N–40°N) (e.g., the Bohai Sea, the southern East China Sea, the southern Japan‐Pacific coastline) and expansion in high latitude regions (40°N–60°N) (e.g., the southern Sakhalin Island, the northern Sea of Japan, the southern Kamchatka Peninsula and the Alaska Peninsula) under future moderate (SSP2‐4.5) and high (SSP5‐8.5) emission scenarios. These results demonstrate that integrating phylogeographic information (e.g., the identification of ancestral persistence areas and unique populations harboring rare allelic variation or high endemism) with SDMs‐based assessments of climate change impacts, offers a powerful framework for prioritizing conservation units in marine benthic ecosystems under future climate change.

## 1 Introduction

One of the top priorities for marine biodiversity conservation and management is to understand and forecast the response of species to climate fluctuation (Bystriakova et al. 2014; Duarte et al. 2018). Climate change has emerged as a major ecological threat, profoundly altering the distribution and abundance of macroalgal ecosystems worldwide (Jueterbock et al. 2013; Martínez et al. 2018; Wilson et al. 2019; Song et al. 2021; Assis, Fragkopoulou, et al. 2024). Facing these global, regional and local climate changes, marine macroalgae must respond via range shifts (facilitated by dispersal) and/or evolutionary adaptation (Harley et al. 2012). In turn, failure or delay in response due to limited dispersal or adaptive capacity increases the probability of local or global range contraction and extinction. These contrasting outcomes (i.e., successful range shifts versus contraction and extinction), leave traceable signatures in the demographic and genetic characteristics of species. Inferring genetic imprints of past climate fluctuations can thus provide critical insights to predict future changes in distribution range and diversity loss due to environmental changes.

Empirically, phylogeography is pivotal for inferring historical climate changes (e.g., glacial–interglacial cycles) that have shaped current geographic distribution of intra‐ and inter‐specific genetic variation and identifying biogeographic events (e.g., migration) that have contributed to lineage divergence (Hu et al. 2011, 2018; Song et al. 2021; Zhang et al. 2025). Episodes in which low genetic diversity coincides with rapid climate change have been linked to increased species extinction risk in ice ages (Hewitt 2004). For marine macroalgae, whose limited dispersal capability is determined largely by water flow (Norton 1992), highly structured genetic diversity can render them more vulnerable to climate change than panmictic species (Leblois et al. 2006). Reliable prediction of species' sensitivity to climate change cannot be inferred solely from the assessment of current distribution ranges and climatic conditions (Thuiller et al. 2005). Instead, integrating factors that control the spatiotemporal genetic variation (e.g., ancestral lineage that survived historical climates and migration‐driven population connectivity) with species distribution models (SDMs), has been argued to improve future biodiversity conservation and management (Blanco‐Pastor et al. 2013; Forester et al. 2013; Bystriakova et al. 2014; Liu et al. 2022; Assis, Fragkopoulou, et al. 2024). Nevertheless, this combined phylogeography‐SDMs framework has rarely been used to infer the past and predict the future for sessile species in the North Pacific (but see Zhang et al. 2019; Song et al. 2021; Liu et al. 2022).

The North Pacific supports exceptionally high marine macroalgal diversity and endemism (Keith et al. 2014). This region, however, has experienced, and continues to experience, marked declines in the geographic range and biomass of macroalgae under the combined impacts of climate change and anthropogenic activities (Tanaka et al. 2012; Song et al. 2021; Liu et al. 2022; Zhou et al. 2023). The annual red macroalga *Gloiopeltis furcata* (Postels & Ruprecht) J. Agardh 1851 is widely distributed along both the western and eastern rocky shores of the North Pacific, where it serves as a foundation species providing food, shelter and breeding grounds for numerous organisms. Exhibiting a distinctly photophilous growth preference with pronounced vertical zonation, *G. furcata* also shows remarkable thermal tolerance and desiccation resistance during daily tidal fluctuation (Liu et al. 2019). Phylogenetic and phylogeographic studies have revealed high lineage diversity in *G. furcata* (Hanyuda et al. 2020; Yang et al. 2020, 2021), with the magnitude of lineage‐level genetic divergence comparable to that observed among species within the Florideophyceae. Consequently, these lineages, together with closely related congeners (e.g., *G. complanate*, *G. tenax* , *G. frutex*, and *G. compressus* ), are treated collectively under a broad *G. furcata* sensu lato (s.l.) concept (Lee et al. 1996).

*Gloiopeltis furcata* s.l. represents a well‐suited model system for incorporating phylogeographic history and projection‐based SDMs to inform marine biodiversity conservation and management in the North Pacific. It combines several key attributes including: (a) a wide distribution range extending from Mashike, Japan (43°51′09″N) to Nanao Island, China (23°26′04.4″N) in the northwest, and from Seal Cove (54°19′57.0″N) to Bamfield, Canada (48°50′20.8″N) in the northeast; (b) regional endemism of distinct genetic lineages; (c) high cryptic genetic diversity; and (d) pontential for long‐distance dispersal facilitated by oceanic currents. Recent genetic studies have delineated multiple diverged lineages and substantial genetic variation within *G. furcata* s.l. (Hanyuda et al. 2020; Yang et al. 2020, 2021), including the identification of potential glacial refugia in Korea and Japan (Yang et al. 2021), the synonymisation of *Gloiopeltis minuta* and *Gloiopeltis dura* with *G. furcata* (Hanyuda et al. 2020), and reinstatement of *Gloiopeltis coliformis* (Kawai et al. 2026). However, these studies lacked a comprehensive coverage of the North Pacific, particularly the inclusion of Chinese populations, resulting in critical knowledge gaps in our understanding of how past climate fluctuations interacted with oceanographic processes to shape the current distribution and genetic lineages of this species.

Although rich lineage diversity indicates that the northwestern Pacific may represent a genetic diversity center for *G. furcata* s.l. (Yang et al. 2021), both quantitative (e.g., coalescent‐based estimation) and predictive (e.g., SDM‐based projection) evidence supporting the hypothesis remain scarce. In this study, we aimed to address these gaps by: (i) identifying populations and/or lineages with high endemism or rare genetic variation across the species' distributional range, (ii) reconstructing the putative area of ancestral persistence during glacial–interglacial cycles and inferring migration routes driven by ocean currents, and (iii) prioritizing conservation units (e.g., populations or lineages) by applying SDMs to predict changes in habitat suitability and associated genetic diversity under future climate scenarios. This integrative phylogeography‐SDMs framework offers critical insights for conserving and managing *G. furcata* s.l. under continuing global change, particularly given that this species has experienced substantial biomass declines driven by ocean warming and overharvesting along parts of the North Pacific coast in China (Xue et al. 2024).

## 2 Materials and Methods

### 2.1 Collection, DNA Extraction and PCR Amplification

**FIGURE 1:**
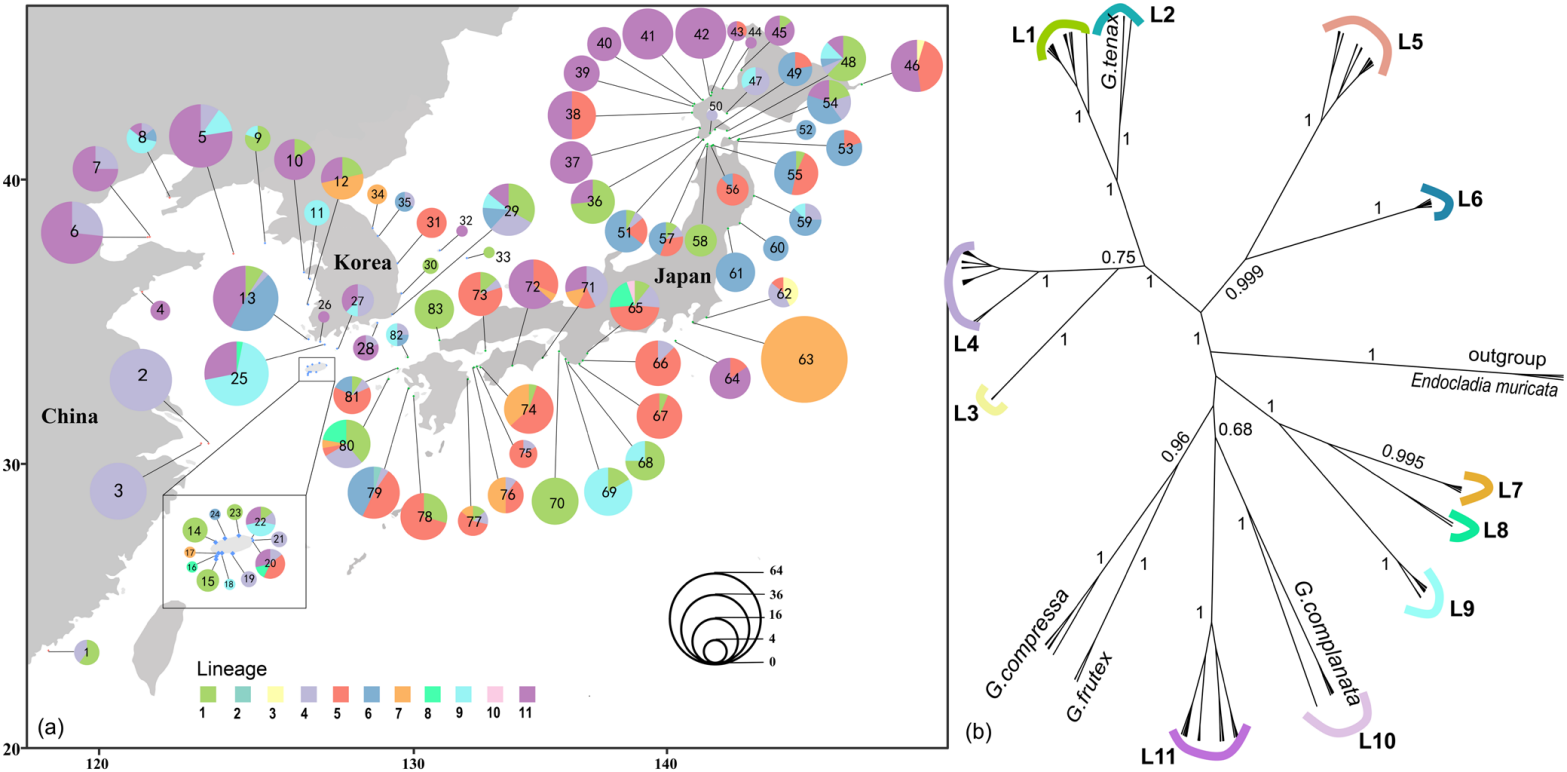
Intraspecific structured lineages of *Gloiopeltis furcata* s.l. inferred from mtDNA *cox*1 haplotypes. Geographic distribution of 11 lineages in the north Pacific (a) and Bayesian inference of structured lineages (b). Numbers on the nodes of clades/lineages represent Bayesian posterior probabilities.

By integrating recently collected specimens from China with previously published datasets (Yang et al. 2020, 2021), we obtained a total of 85 populations of *G. furcata* s.l., of which eight were newly collected from China (Figure 1, Tables S1 and S2). Populations were assigned to different geographic regions following the classification scheme of Yang et al. (2021) (Tables S1 and S2). To minimize sampling of identical genotypes, an inter‐individual spacing of > 10 m at each site was maintained. After collection, thalli were air‐dried and preserved with silica gel. Genomic DNA was extracted using the method described by Hu et al. (2004). A fragment of the mitochondrial *cox*1 gene was amplified using the newly designed primers cox‐F (5′‐CCAATCACAAAGACATAGG‐3′) and cox‐R (5′‐GCCAAAGAATCAGAAAA‐3′), with PCR conditions consisting of an initial denaturation at 96°C for 4 min; 35 cycles of 30 s at 96°C, 30 s at 45°C, and 1 min at 72°C; and a final extension at 72°C for 7 min. A fragment of the chloroplast *rbc*L gene was amplified using two primer pairs rbcl‐F (5′‐ACAAGAATCAAAGACGAA‐3′) and rbcl‐R (5′‐TTACAGATAACACGGAAAT‐3′) and F762 (5′‐GTATGAAAGAGCTGAATTTGC‐3′) and R1442 (5′‐AAACATTAGCTGTTGGAGTTTCTAC‐3′) (Kim et al. 2010), with conditions of 4 min at 96°C; 35 cycles of 1 min at 96°C, 1 min at 45°C, and 2 min at 72°C; and a final extension at 72°C for 7 min.

MtDNA *cox*1 and cpDNA *rbc*L were each sequenced in both directions following the methods of Yang et al. (2020, 2021), using an Applied Biosystems 3730XL at Bioengineering Co. Ltd. (Shanghai, China). Sequences were aligned and manually edited in MEGA v11.0.11 (Tamura et al. 2021), yielding 971 *cox*1 sequences, of which 147 were newly generated (Table S1) and 336 *rbc*L sequences, of which 121 were newly generated (Table S2).

### 2.2 Phylogeographic Diversity and Lineage Structuring

After alignment and edition, we obtained 567 bp of *cox*1 and 1223 bp of *rbc*L for *G. furcata* s.l. Phylogeographic diversity parameters—the number of haplotypes (*N*_*h*_), haplotype diversity (*h*), and nucleotide diversity (*π*) – were estimated in Arlequin v3.5.1.2 (Excoffier and Lischer 2010). Pairwise genetic differentiation (*F*_ST_) was calculated in Arlequin with significance assessed by 10^5^ permutations. Analysis of molecular variance (AMOVA) was conducted in Arlequin with 10^4^ permutations to partition genetic variation within populations, among populations within groups, and among groups based on different grouping criteria such as country, longitude, marine ecoregion, marine province, and biogeographic basin (Tables S3 and S4).

Lineage structure was firstly assessed according to haplotype phylogeny, which was reconstructed using Bayesian inference (BI) in BEAST v1.10.4 (Suchard et al. 2018), with the red macroalga *Endocladia muricata* chosen as outgroup. The best‐fit nucleotide substitution model was selected using ModelFinder in PhyloSuite v1.2.3 (Zhang, Gao, et al. 2020) under the Akaike information criterion (AIC). In BEAST, the tree prior was set to a speciation (Yule) process with a strict molecular clock. Markov chain Monte Carlo (MCMC) analyses were run for 1 × 10^9^ steps under a GTR + G model, sampling every 10^4^ steps and discarding the first 2 × 10^8^ steps as burn‐in. Convergence and mixing were assessed in Tracer v1.7.1 (Suchard et al. 2018), considering effective sample sizes (ESS) > 200 as an indicative of convergence. A maximum clade credibility tree was summarized in TreeAnnotator v1.7.5 (Suchard et al. 2018) after removing the first 10% of trees as burn‐in, and visualized in FigTree v1.4.3. Lineage structuring was further examined through a minimum spanning tree (MST) computed in Arlequin.

### 2.3 Historical Gene Flow and Reconstruction of Ancestral Distribution

To determine whether long‐distance dispersal of *G. furcata* s.l. contributed to phylogeographic structuring and temporospatial distribution patterns, historical gene flow among regions and populations was estimated with Migrate‐n v5.0.4 (Beerli et al. 2019). MCMC was run following the method described by Li et al. (2017). Putative ancestral distribution of *G. furcata* s.l. in the North Pacific was reconstructed in RASP v4.3 (Yu et al. 2020) using the BEAST tree set. Based on the geographic distribution of *G. furcata* s.l. in the North Pacific, four biogeographic regions were defined: **A**—Northern Japan (HOK, THK) and Far East Russia (KAM), **B**—Southern Japan (KAT, SKJ, KAS, SKK, KYS) and Korea (JJK, SOK, EAK), **C**—China (GZ, ZJ, SD, LN) and Korea (WEK), and **D**—Canada (BCC). Ancestral states were inferred under the S‐DIVA (Statistical Dispersal–Vicariance Analysis) framework, and the possible ancestral range at each node and the most probable scenarios were retained. To account for uncertainties in phylogeny, we used 9000 trees from the MCMC output and performed S‐DIVA on all of them (Ali et al. 2011). These two phylogeographic analyses focused primarily on the *cox*1 dataset because it provided richer phylogeographic information and broader geographic coverage than *rbc*L.

### 2.4 Species Distribution Prediction

Occurrence records of *G. furcata* s.l. in the North Pacific were compiled based on published literature (Table S5) and sampling localities (Table S1), online resources, and public databases (AlgaeBase: www.algaebase.org; Global Biodiversity Information Facility: http://www.gbif.org; iNaturalist: https://www.inaturalist.org; iDigBio: https://www.idigbio.org; Atlas of Living Australia: https://www.ala.org.au; Ocean Biogeographic Information System: http://iobis.org). In total, 3339 records were retrieved. To ensure data quality, we scrutinized and cleaned the occurrences by (i) removing erroneous records falling outside the species' natural range (e.g., terrestrial points), and (ii) reducing spatial clustering by retaining a single record within each 3‐arc‐minute grid cell. The final dataset comprised 338 unique, quality‐controlled records in the North Pacific (Figure S1).

Given that *G. furcata* s.l. typically grows in the upper intertidal and supratidal zones by attaching to shallow rocky substrates, we retrieved 16 sea‐surface environmental layers and one bathymetric layer from the Bio‐ORACLE v3.0 database (https://bio‐oracle.org) (Assis, Fernández, et al. 2024), each at a spatial resolution of 3 arc‐minutes. To reduce the adverse effects of multicollinearity, we followed Dormann et al. (2013) and computed pairwise Pearson correlation coefficients among predictors. Variables with |*r*| > 0.7 were considered highly correlated (Figure S2), and within each such set we retained the predictor with the strongest ecological interpretability to avoid model overfitting (Solstorm et al. 2015). Because *G. furcata* s.l. is tolerant to high irradiance, aerial exposure, and desiccation, we ultimately selected seven ecologically meaningful and mutually independent predictors (Table 1): sea surface temperature‐maximum (SST max), sea surface temperature‐minimum (SST min), sea surface temperature‐range (SST range), sea surface salinity mean (SSS mean), depth (BD), distance to shore, and current velocity mean (CV mean). In accordance with the Sixth Assessment Report (AR6) of the Intergovernmental Panel on Climate Change (IPCC, 2021), we adopted the CMIP6 Shared Socioeconomic Pathways (SSPs) scenarios SSP2‐4.5 (intermediate emission) and SSP5‐8.5 (high emission), and extracted from Bio‐ORACLE the corresponding future projection layers for the five key environmental predictors for downstream analyses.

**TABLE 1:** The contribution of different environmental variables to habitat suitability of *G. furcata* sensu lato.

Marine environmental variables	Contribution (%)
Distance to shore	88.5
Sea surface temperature‐minimum (SST min)	6.1
Depth (BD)	4.3
Sea surface temperature‐maximum (SST max)	0.2
Sea surface temperature‐range (SST range)	0.4
Current velocity mean (CV mean)	< 0.1
Sea surface salinity mean (SSS mean)	0.5

We then implemented MaxEnt for model building. First, we delineated the study extent by creating a 500‐km buffer around the occurrence records. Within this extent, we randomly generated 10,000 background points (Thuiller et al. 2009; Guisan et al. 2017). Model tuning targeted two parameters—the regularization multiplier (RM) and feature classes (FC). RM values ranged from 0.5 to 4.0 in 0.5 increments (eight levels), and FC comprised four settings: linear (L), quadratic (Q), hinge (H), and a combined class, yielding 32 RM‐FC combinations in total. Predictive performance for each combination was assessed with the ENMeval R package (Kass et al. 2021) and ranked using the AIC. In addition, we evaluated model performance with a fivefold cross—validation design repeating 10 times and randomly assigning 80% of occurrences for training/tuning and 20% for internal testing (Muscarella et al. 2014).

Performance of the final models was evaluated using the area under the receiver operating characteristic curve (AUC) (Swets 1988), the True Skill Statistic (TSS) (Allouche et al. 2006), and the continuous Boyce index (Hirzel et al. 2006). The TSS jointly considers sensitivity, specificity and explicitly accounts for both omission and commission errors (Anderson 2003; Allouche et al. 2006), with values in [−1, 1]: TSS ≥ 0.8 indicates excellent performance. AUC ranges from 0 to 1; by convention, AUC ≥ 0.9 suggests outstanding performance (Anderson 2003). We considered models with AUC ≥ 0.7, TSS ≥ 0.4, and Boyce ≥ 0.4 to have acceptable predictive performance (thresholds) (Araújo et al. 2005). Continuous suitability outputs were converted to binary habitat suitability maps using a 10% training—omission threshold (Lobo et al. 2010), that is, a cutoff chosen to ensure that ≤ 10% of training occurrence points were misclassified, thereby balancing model errors. We then transformed the binary habitat suitability projections of binary habitats suitability to the Lambert Cylindrical Equal Area projection at a resolution of 10 km and estimated the areas of potential distribution (Zhang, Capinha, et al. 2020).

## 3 Results

### 3.1 Phylogenetic Diversity and Lineage Structuring

**FIGURE 2:**
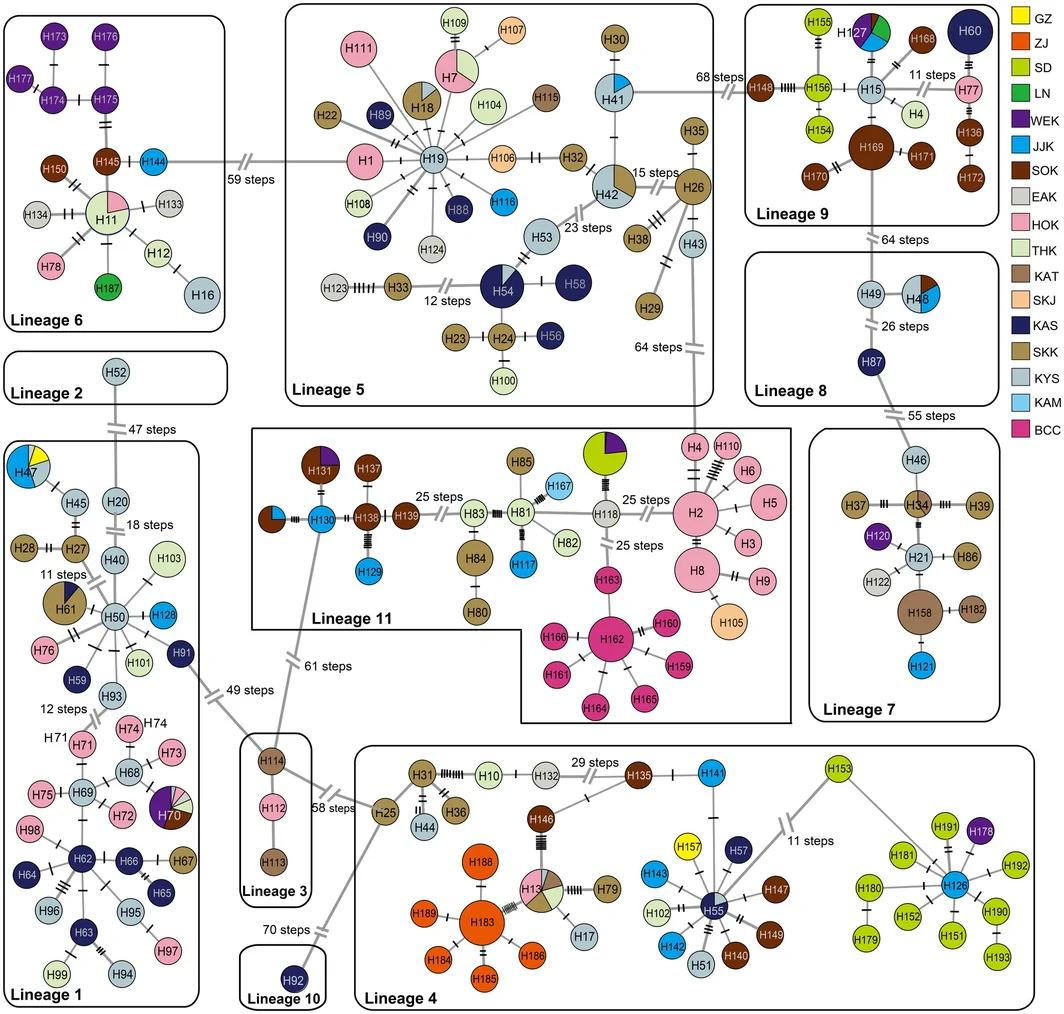
MtDNA *cox*1 haplotype network in *Gloiopeltis furcata* s.l. Circle size is proportional to population sample size. Each line between haplotypes represents one mutation step. BCC: British Columbia, Canada; EAK, East coast of Korea; GZ: Guangzhou, China; HOK, Hokkaido, Japan; JJK, Jeju Island, Korea; KAM: Kamchatka, Russia; KAS, Kansai, Japan; KAT, Kanto, Japan; KYS, Kyushu, Japan; LN: Liaoning, China; SD: Shandong, China; SKJ, Shikinejima, Japan; SKK, Shikoku, Japan; SOK, South coast of Korea; THK, Tohoku, Japan; WEK, West coast of Korea; ZJ: Zhejiang, China.

971 mtDNA *cox*1 comprised 217 variable sites, yielding 193 haplotypes, of which 20 were newly generated (H151‐H157, H179‐H180, H183‐H193) (Table S1). Among the 193 haplotypes, 23 occurred in China, 50 in Korea, 117 in Japan, one in Russia and eight in Canada (Figure 2, Table S1); 176 occurred in a single population and 105 were singletons (Figure 2). Haplotypes H70 and H13 exhibited the widest distributional ranges, spanning multiple geographically separated localities. Overall, *G. furcata* s.l. across the North Pacific harbored high haplotype diversity (*h* = 0.9721). Among the 85 populations sampled, only 29 exhibited *h* values lower than 0.45 (Table S1). The Shizuura population from Hokkaido, Japan exhibited the highest nucleotide diversity (*π* = 0.004667). Comparatively high nucleotide diversity was also observed in other populations from Hokkaido (mean *π* = 0.000519) and the west coast of Korea (mean *π* = 0.000449) (Table S1). 336 cpDNA *rbc*L sequences identified 49 haplotypes, of which 11 were newly generated (H2‐H6, H8‐H13) (Table S2), and revealed lower haplotype and nucleotide diversity than mtDNA *cox*1 (Tables S1 and S2). All newly generated *cox*1 and *rbc*L haplotypes have been deposited in GenBank under accession numbers PZ547756‐PZ547786.

Across the 10 alternative grouping criteria, *cox*1‐based AMOVA consistently showed that 49%–63% of the total genetic variance occurred among populations within groups, followed by levels of within‐population variance (24%–26%) and among groups (mean value = 19%; Table S3). The cpDNA *rbc*L dataset further indicated that variance among populations within groups accounted for 70%–87% of the total genetic variance (Table S4).

Bayesian phylogenetic analysis delimited 193 *cox*1 haplotypes into 11 lineages (L1–L11) with high posterior probabilities (PP: 0.995–1.000) (Figure 1b). The MSN‐based network also clustered the 193 haplotypes into 11 lineages differing by ≥ 45 bp (Figures 1, 2, Table S6). Geographically, L1 predominates along the southwestern coasts of Korea and the Tsugaru Strait, Japan. L2 consists of a single haplotype H52 and is restricted to Kayaki, Kyushu, Japan. L3 is narrowly endemic to Habomai, Hokkaido, and Katsuura, Kanto, Japan. L4 is widely distributed along the coastlines of China, Korea, and Japan. L5 is absent from China and mostly occurs predominantly in Japan (e.g., Tohoku, Kansai, Shikoku and Kyushu). L6 is concentrated in Hokkaido and Tohoku, Japan. Lineages 7–9 show a scattered distribution across the North Pacific. L10 consists of a single haplotype (H92) and is confined to Mie, Kansai, Japan. L11 is most frequent in the Yellow‐Bohai Sea, the Tsushima Strait, Hokkaido. Kamchatka, Russia and British Columbia, Canada (BCC) each harbor only L11. Southwestern Japan (i.e., Kansai, Shikoku and Kyushu) harbors 10 lineages (L1–L2, L4–L11), whereas the southern Korea (i.e., the west coast and the Jeju Island) harbors eight lineages (L1, L4–L9, L11) (Table S6). The Chinese coastline harbors five lineages (L1, L4, L6, L9, L11), of which L1 is restricted to Guangdong and L4 to Liaoning. L9 occurs in both Shandong and Liaoning, and L4 occurs across the coastlines of China (Figure 1a, Table S6).

*rbc*L haplotype clusters were operationally delimited as lineages differing by ≥ 30 bp, yielding nine genetic lineages (A‐I) (Figures S3 and S4), which broadly, though not exactly, corresponded to the *cox*1‐derived rich cryptic lineages in *G. furcata* s.l. across the North Pacific. The Chinese coastline is solely dominated by lineage G, Japanese coastlines are characterized by lineages D, E, and I, whereas the southern coast of Korea harbors seven lineages (A, B, C, D, F, G, H) (Figure S4).

### 3.2 Gene Flow and Ancestral Distribution

**FIGURE 3:**
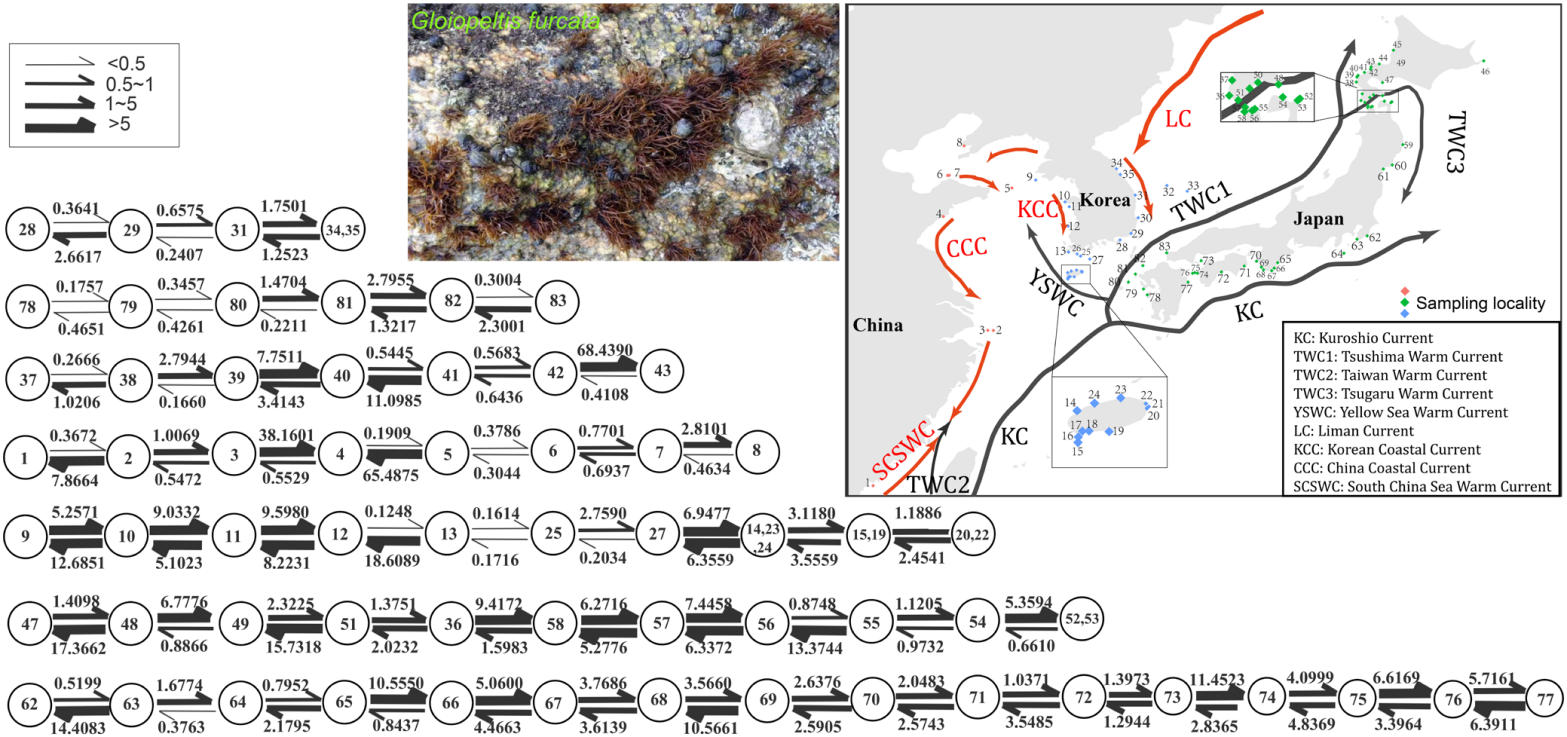
Estimates of historical gene flow (*N*_m_) between populations of *Gloiopeltis furcata* s.l. in north Pacific. Numbers above/below arrows represent potential migration rates in the direction of the arrow. The thickness of arrow is scaled according to the values. Numbers in the circles represent population locations as shown in Figure 1 and Table S1.

MtDNA *cox*1 revealed complex historical genetic exchange among *G. furcata* s.l. populations across the North Pacific. Overall, bidirectional migration rates were observed at both region and population levels (Figure 3; Figure S5). For geographically proximate populations such as those on both sides of the Tsushima Strait, along the coast of China, and on the Japan‐Pacific coast, continuous and comparable bidirectional migrations were detected among adjacent populations (Figure 3). Historical gene flow was particularly pronounced along the west coast of Korea and in the Tsugaru Strait, Japan (Figure 3).

**FIGURE 4:**
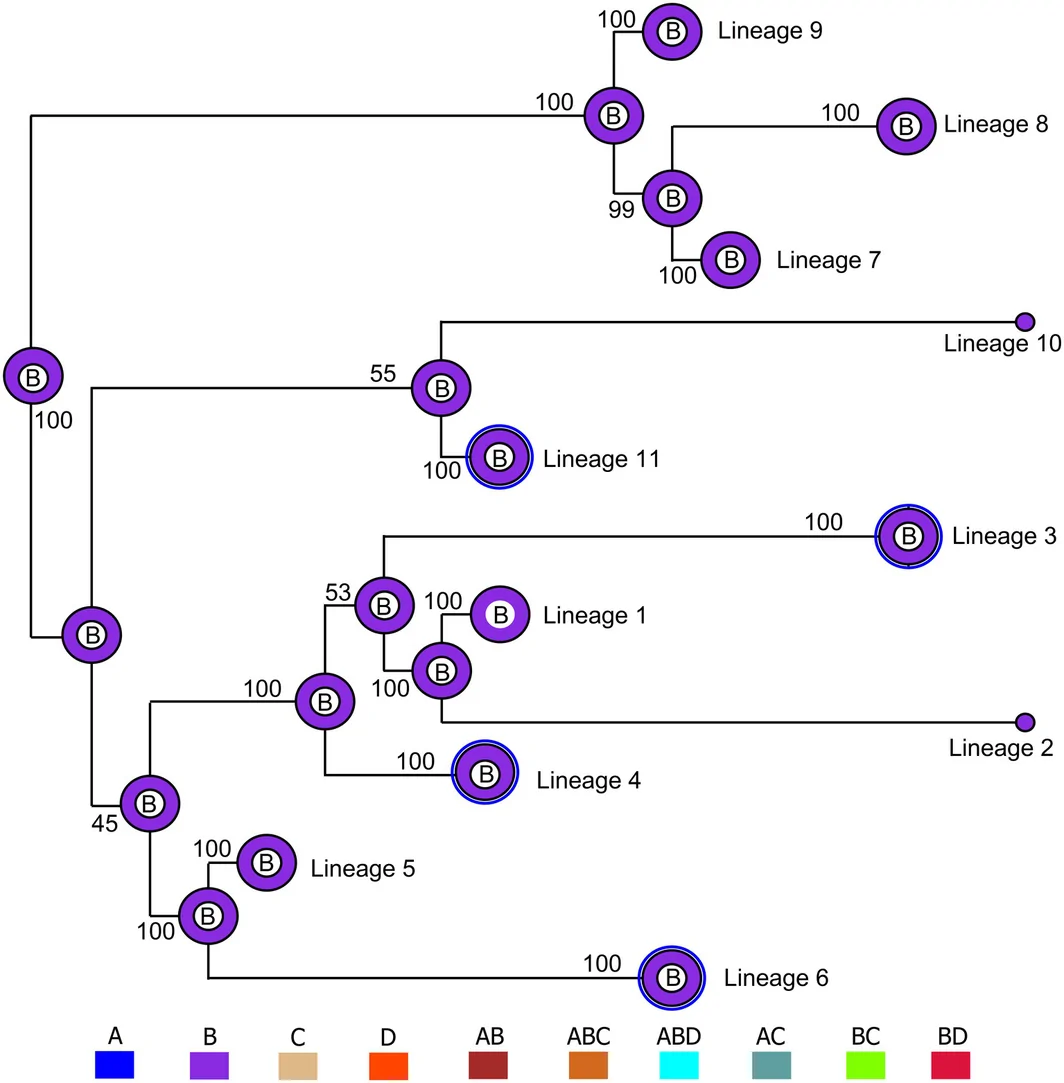
Ancestral distributional reconstruction of mtDNA *cox*1 lineages in *Gloiopeltis furcata* s.l. from S‐DIVA (RASP). Color key to possible ancestral ranges at different nodes, and the blue circles outside of the node represent dispersal events. Numbers on the nodes represent Bayesian posterior probabilities. Biogeographical regions: (A) Northern Japan (HOK, THK) and Russia (KAM); (B) Southern Japan (KAT, SKJ, KAS, SKK, KYS) and Korea (JJK, SOK, EAK); (C) China (GZ, ZJ, SD, LN) and Korea (WEK); (D) Canada (BCC).

S‐DIVA revealed a complex biogeographical history of *G. furcata* s.l. in which 48 dispersal events, 34 vicariance events, and a single extinction played key roles in shaping the present‐day distribution in the North Pacific (Figures 4 and S6). Lineage 1 exhibited the highest frequencies of both dispersal (*n* = 10) and vicariance (*n* = 8). Dispersal events were most frequent in southern Japan and the adjacent Tsushima Strait (*n* = 32), whereas vicariance events occurred mostly in southern Japan and the adjacent Jeju Island, the south and east coasts of Korea (*n* = 23) (Figure S6). S‐DIVA inferred a single ancestral range of *G. furcata* s.l., corresponding to area **B** (southwestern Japan and southern Korea) with 100% Bayesian support value (Figure 4). Lineage level S‐DIVA reconstructions likewise indicated that all lineages originated in southwestern Japan and southern Korea (Figure S6). Subsequent dispersal and vicariance events resulted in highly structured sub‐lineages within lineages 1, 4, 5, 6, 9, and 11 (Figure S6).

### 3.3 Model Performance and Projected Habitat Suitability

MaxEnt projections of geographic distribution yielded strong discriminatory performance (AUC = 0.986, TSS = 0.911, Boyce = 0.968) all of which exceeded their respective thresholds, indicating high confidence in the model's habitat suitability predictions in the North Pacific. Among the seven key environmental variables, distance to shore was identified as the dominant driver of habitat suitability (88.5%), followed by minimum SST (6.1%) and bathymetric depth (4.3%) (Table 2). SSS mean and CV mean had comparatively minor effects.

**TABLE 2:** The change of distribution range of *G. furcata* sensu lato under different greenhouse gas emission scenarios.

Time period	SSP	Current range size (× 10^6^ km^2^)	Loss (%)	Gain (%)	Range size change (%)
2040–2050	SSP2‐4.5	1.4	−4.9	4.7	−0.2
	SSP5‐8.5	1.4	−3.7	6.7	3.0
2090–2100	SSP2‐4.5	1.4	−5.0	19.0	14.0
	SSP5‐8.5	1.4	−11.1	45.9	34.8

Under present‐day conditions, the continuous model predicted that suitable habitats were distributed in the North Pacific between 20°N/60°N and 120°E/120°W (Figure S7a). The areas of highest suitability were centered on the northern Gulf of Alaska southward to California, USA in the east and Hokkaido, Japan southward to northern Guangdong, China, in the west. The binary prediction delineated a suitable range largely congruent with the continuous output (Figure S7b), reinforcing the robustness of the modeled present‐day distribution.

**FIGURE 5:**
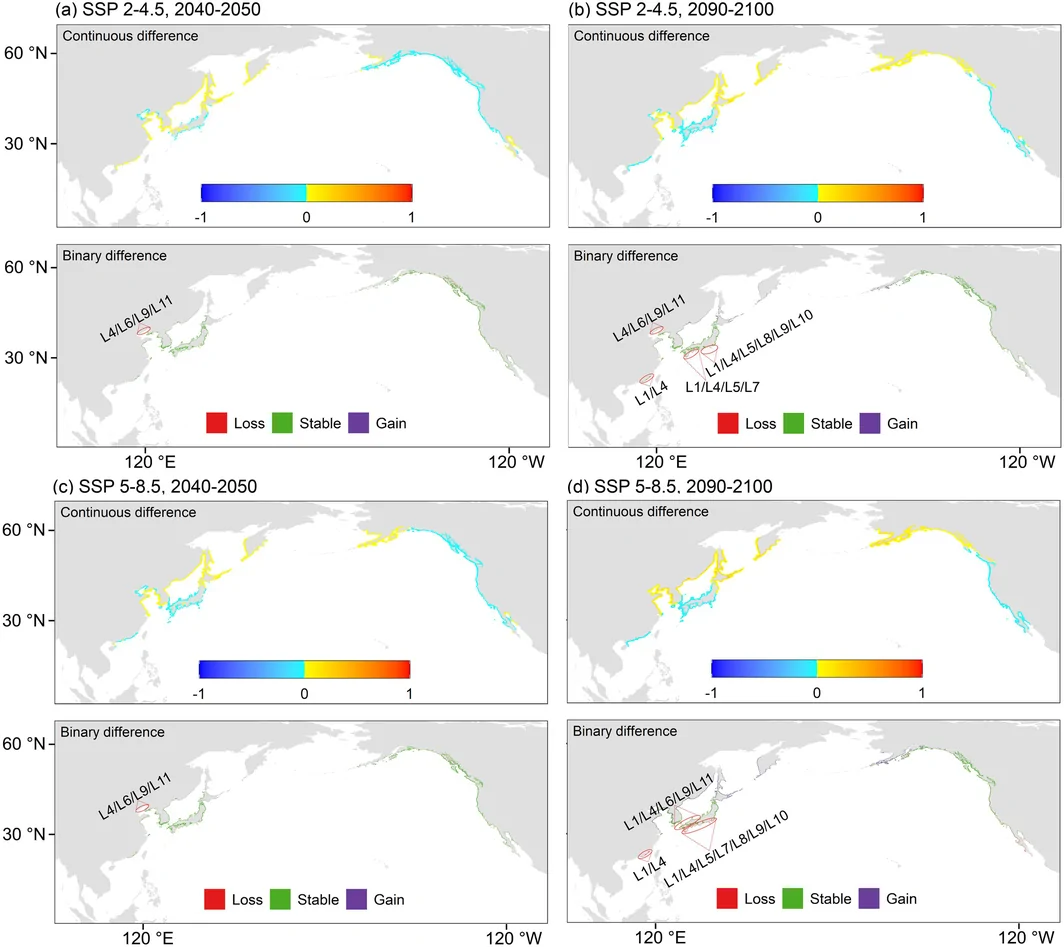
Changes in habitat suitability of *Gloiopeltis furcata* s.l. by the 2040s and 2090s under the SSP2‐4.5 (a, b) and SSP5‐8.5 (c, d) scenarios, respectively. Red ellipses represent projected lost suitable habitats and associated genetic lineages.

Under the SSP2‐4.5 and SSP5‐8.5 scenarios, projections consistently indicated contraction of habitat suitability at low‐to‐mid latitudes and expansion at high latitudes (Figure 5), indicating a poleward shift in potential range of *G. furcata* s.l. Under the SSP2‐4.5, habitat suitability remained broadly stable during the 2040s (2040–2050), with visible gains in the northern Sea of Japan (40°N–45°N) and losses in the northern Bohai Sea (Figure 5a). By the 2090s (2090–2100), suitable habitats were further lost or fragmented in the southern East China Sea, the northern Bohai Sea, and the southern Japan‐Pacific margin (Figure 5b). Meanwhile, new suitable habitats emerged in the northern Sea of Japan, the southern Sakhalin, the southern Kamchatka, the eastern Hokkaido, and the Alaska Peninsula (Figure 5b). Under the SSP5‐8.5, the patterns of gain and loss of habitat suitability by the 2040s were similar in direction but greater in magnitude than those under SSP2‐4.5 (Figure 5c,d). By the 2090s, suitable habitat in the East China Sea (i.e., the Guangdong province) had disappeared completely, whereas the Yellow and Bohai Seas re‐gained habitat suitability. Suitable habitats along the southern Japan‐Pacific margin and the southern Sea of Japan became substantially lost or fragmented. A comparable pattern of habitat loss was also projected for Baja California, Mexico (Figure 5d). The potential gain in habitat suitability resembled the pattern projected under SSP2‐4.5 (Figure 5d), with northward expansion along high‐latitude coasts.

Under SSP2‐4.5, *G. furcata* s.l. in the North Pacific is projected to experience a 0.2% contraction in habitat range (*c*. 2.80 × 10^3^ km^2^) relative to its current distribution by the 2040s. By the 2090s, however, the net habitat suitability is projected to increase significantly, leading to a 14.0% expansion relative to the current range (Table 2). Under SSP5‐8.5, the magnitude of range expansion is predicted to increase substantially, leading to a significant net expansion in habitat suitability (3.48 × 10^5^ km^2^) by the 2090s (Table 2), consistent with the stronger warming signal in this scenario.

## 4 Discussion

In this study, the inclusion of Chinese populations of *G. furcata* s.l. did not yield new lineages, but added unique haplotypes (Figures 1 and S4, Tables S1 and S2) in comparison to previous studies (Hanyuda et al. 2020; Yang et al. 2020, 2021). These haplotypes (i.e., mtDNA *cox*1) are assigned to lineages L1, L4, L6, L9 and L11, which also occur widely in southwestern Japan and southern Korea (Figure 1a, Table S6). Such haplotype endemism and distributional patterns along the coast of China, together with the RASP results (Figure 4), indicate that the most recent common ancestor of *G. furcata* s.l. likely originated in the southern Japanese Archipelago and adjacent Korean waters (Figure 4), a region that has harbored multiple glacial refugia throughout past glacial–interglacial cycles. This phylogeographic insight essentially aligns with previous reports on the role of Quaternary climatic shifts on the distribution of benthic seaweeds in the North Pacific (Hu et al. 2011, 2015, 2018; Ng et al. 2019; Liu et al. 2022; Wang et al. 2025).

Paleogeographical reconstructions illustrated that glacial maximum induced sharp cooling and sea‐level fall that exposed marginal basins in the North Pacific (e.g., the Yellow‐Bohai and Japan Seas, Wang 1999), potentially led to the ancestral relic lineages of *G. furcata* s.l., to survive in multiple glacial maximum (i.e., long‐term refugia), and then fragmented the distribution range of *G. furcata* s.l. Geographically, this tectonic reconfiguration resulted in relic lineages of *G. furcata* s.l. to persist in separate, isolated refugia in the North Pacific as proposed in other benthic seaweeds (Hu et al. 2011; Kim et al. 2012; Li et al. 2017, 2020; Song et al. 2021). Following deglaciation during interglacial period, sea‐level rise and the reopening of straits (e.g., the Tsushima Strait) reconnected marginal Yellow‐Bohai, Japan and East China seas (Wang 1999), enabling geographically isolated ancestral populations of *G. furcata* s.l. to expand and colonise their ranges along coastlines and consequently lineage admixture (Hu et al. 2011; Ni et al. 2014).

Our historical gene flow patterns (Figure 3) suggest that regional current systems likely contributed to postglacial range expansion, ultimately shaping population genetic connectivity, lineage diversity and distribution of *G. furcata* s.l. across the North Pacific (Hu et al. 2011; Ni et al. 2014; Li et al. 2017, 2020). These ocean current systems not only drove lineage‐level genetic exchange (Figure 3) and created contact zones with admixed genetic variation, but also can potentially promoted diversification and speciation over time (Hewitt 2004). Indeed, glaciation‐induced geographic isolation between the Yellow–Bohai and East China Seas generated substantial genetic divergence among lineages, which was subsequently attenuated by Kuroshio‐derived dispersal corridors near Jeju Island (Li et al. 2017). In particular, the northward‐flowing Kuroshio Current system (Figures 3 and S5) not only facilitated the poleward expansion of benthic sessile species such as *G. furcata* s.l., but also promoted long‐distance dispersal of seaweed propagules among higher‐latitude populations (Hu et al. 2011; Li et al. 2017, 2020; Song et al. 2021). This current‐driven biogeographic process thus explain how *G. furcata* s.l. expanded from the glacial survival center around the southwestern Japan and southern Korea to its current range‐wide distribution in the North Pacific (Figures 1 and S4, Table S6).

Herein, distance to shore was identified as the most important environmental predictor for *G. furcata* s.l. in the North Pacific (Table 1). Distance to shore often co‐varies and interacts with water depth, leading to marked spatial variation in macroalgal assemblage composition both among depths and along nearshore‐offshore gradients (Webber et al. 2022). In this context, climate‐driven increases in SST and sea‐level rise can significantly alter nearshore habitat configuration (e.g., effective distance to shore, depth and exposure), thereby not only reshaping macroalgal distribution patterns (e.g., range shifts and localized extirpations), but also imposing strong constraints on physiological performance and reproduction (e.g., photosynthetic efficiency and reproductive phenology) (Molinos et al. 2016; de Bettignies et al. 2018). Physiologically, the distinctive tubular thallus and weak water‐holding capacity (Liu et al. 2019) render *G. furcata* s.l. highly susceptible to environmental fluctuations, as its growth can be regulated by temperature, irradiance, and desiccation. For example, spore germination of *G. furcata* s.l. in Guangdong, China is optimal at 10°C–20°C in the wild (Chen et al. 2011). However, summer SST exceeding 23°C can cause the thallus degradation (Wu et al. 2007). Again, *G. furcata* s.l. in laboratory can reach the optimal growth at temperatures between 10°C–14°C (Chen, Chen, Zhu, Guo, and Guo 2014), but its photosynthetic performance can be significantly inhibited by concurrent high irradiance (1000 μmol photons m^−2^ s^−1^) and high temperature (Liu et al. 2014). Aerial exposure for 2–3 h at 26°C–28°C can even result in bleaching and massive mortality (Chen, Chen, Zhu, and Su 2014). These lines of evidence show that climate‐induced changes in SST and associated sea‐level variation (e.g., distance to shore, water depth and emersion regime) can exert considerable stresses on *G. furcata* s.l., ultimately altering its distribution range.

Our SDMs projections of *G. furcata* s.l. forecast significant expansions of habitat suitability at high latitudes in the North Pacific, far exceeding contractions at low latitudes (Figure 5, Table 2). These range shift patterns are broadly congruent with findings reported for other seaweeds (Song et al. 2021; Zhou et al. 2023). For example, the brown macroalga *Sargassum thunbergii* (Mertens ex Roth) Kuntze expanded its range from lower to higher latitudes in the North Pacific since the last glacial maximum, and its habitat suitability is projected to become highly fragmented along the coastlines of China and severely reduced along the Korean Peninsula by the 2090s (Song et al. 2021). Similarly, the red macroalga *Gracilaria vermiculophylla* is projected to lose all habitat suitability in southern Taiwan by the 2090s, alongside the gain of new high‐latitude habitats in the northern Kamchatka Peninsula and the entire Okhotsk Sea (Liu et al. 2022). These asymmetric shifts between the southern and northern range limits entrench the need for preserving and managing benthic seaweed resources in the North Pacific under future climate scenarios.

In the northern Bohai Sea, suitable habitats of *G. furcata* s.l. are projected to be lost entirely by the 2090s under both the SSP2‐4.5 and SSP5‐8.5 scenarios (Figure 5), with the potential consequent loss of four mtDNA *cox*1 lineages (L4, L6, L9, L11). A comparable scenario is predicted for Guangdong, China, where two *cox*1 lineages (L1, L4) currently occupying small and fragmented habitats (field observation) are projected to lose their entire distribution range in the South China Sea. These certain areas with high lineage diversity and endemism warrant particular conservation attention in the face of changing climate (Dawson et al. 2011; Médail and Baumel 2018). Furthermore, the projected habitat loss of *G. furcata* s.l. by the 2090s could potentially lead to local disappearance of massive lineages in the southern Japanese Archipelago (i.e., L1, L4, L5, L7‐L10) and the southwestern Sea of Japan/Tsushima strait (i.e., L1, L4, L6, L9, L11) (Figure 5). These genetically divergent, adaptively distinct, and widely distributed lineages, may each be recognized as an independent evolutionarily significant units (ESUs) warranting in situ conservation (Zhang et al. 2018; Mamo et al. 2021). In contrast, coordinated ex‐situ conservation measures can be implemented for those lineages, which are either concentrated in a specific area (i.e., L1, L4‐L9, L11 in Jeju Island) or geographically isolated from others with small population sizes (e.g., L2 in Kayaki, Kyushu, L3 in Habomai, Hokkaido and Katsuura, Kanto (Figure 1, Table S6)), to sustain their distribution range and associated ecosystem functions under ongoing climate change (Van Rossum et al. 2018; Mamo et al. 2021; Hu et al. 2026). Defined within a comparative phylogeographic framework, areas for climatic persistence (marine refugia), diversification (evolutionary cradles) and dispersal (mediated by physical barriers or corridors such as Jeju Island; Li et al. 2017) by incorporating more co‐distributed benthic species may be pivotal for regional level of conservation planning (Lexer et al. 2013; Avise et al. 2016). It should be noted that a region that served as a long‐term persistence center during the slow environmental changes of ice ages may not indicate future resilience under accelerated climate warming if local conditions exceed physiological tolerance of *G. furcata* . Integrating phylogeography and SDMs with evolutionary diversity metrics (e.g., phylogenetic diversity and endemism), can provide crucial insights into spatial congruence across different taxonomic units and support the building of evolutionary resilience across the North Pacific in the face of increasing climate change and anthropogenic pressures (Sgro et al. 2011; Forester et al. 2013).

In summary, the southwestern Japan and southern Korea served as the center of in situ glacial persistence for *G. furcata* s.l. lineages, highlighting its high conservation value in the North Pacific. Climate change is projected to progressively induce shifts in distribution range, habitat suitability, and associated genetic characteristics of *G. furcata* s.l. across the North Pacific, highlighting urgent concerns for its biodiversity conservation. Integrating phylogeography‐based lineage delimitation, reconstruction of ancestral distribution, and genetic dynamics with SDMs‐projected range shifts, presents an effective approach to developing robust and long‐term conservation efforts for *G. furcata* s.l. To better understand whether and how genetic diversity enhances the resilience of *G. furcata* s.l. and associated communities to a changing climate, a genomics‐informed framework, including the detection of climate‐adapted variants and assessment of adaptive potential, is necessary to resolve conservation and ecosystem services trade‐offs (Zhang et al. 2018; Heuertz et al. 2023). In addition, fully characterizing the hidden lineage diversity of *G. furcata* s.l. and formally raising candidate lineages to species status could help achieve an integrative framework that explicitly combines evolutionary and ecological information, thereby bridging application gap between phylogeography and ES management (Mamo et al. 2021; Heuertz et al. 2023).

## Funding

This work was supported by the National Natural Science Foundation of China, 32371697 (32411540227), start‐up funds from the Outstanding Talent Program at Yantai University, and the development fund of South China Sea Institute of Oceanology of the Chinese Academy of Sciences (SCSIO202208).

## Conflicts of Interest

The authors declare no conflicts of interest.

## Supporting information


**Table S1:** MtDNA‐*cox*1 inferred genetic diversity indices of *Gloiopeltis furcata* sensu lato in the North Pacific.
**Table S2:** CpDNA‐*rbc*L inferred genetic diversity indices of *Gloiopeltis furcata* sensu lato in the North Pacific.
**Table S3:** MtDNA‐*cox*1 based Analysis of Molecular variance (AMOVA) for *G. furcata* sensu lato in the north Pacific using multiple grouping criteria.
**Table S4:** CpDNA‐*rbc*L based Analysis of Molecular variance (AMOVA) for *G. furcata* sensu lato in the north Pacific using multiple grouping criteria.
**Table S5:** Occurrence records of the red seaweeds *G. furcata* sensu lato in the north Pacific from literatures used for species distribution modelling (SDM).
**Table S6:** Distribution of mtDNA‐*cox*1 inferred lineages (L1‐L11) of *G. furcata* sensu lato in each population. Numbers in shaded indicate haplotypes in a specific lineage either distributed proximately (in bold) or endemic in 1–2 populations (in red).
**Figure S1:** 338 filtered occurrence records of the red macroalga *G. furcata* sensu lato in the north Pacific (we only consider regions shallower than 10 m).
**Figure S2:** Correlation levels between marine predictors. We computed the pairwise Pearson's correlation coefficient (*r*) between predictors. Predictors with |*r*| > 0.7 are deemed high correlated.
SST: sea surface temperature; CV: current velocity; SSS: sea surface salinity; DO: dissolved oxygen; Bd: seawater depth; max: annual maximum value; mean: annual mean value; min: annual minimum value; range: annual range.
**Figure S3:** Parsimony median‐joining network in the red seaweeds *G. furcata* sensu lato based on cpDNA‐*rbc*L haplotypes, which identified 9 lineages (A‐I). Circle size is proportional to population size, and each line between haplotypes represents one mutation step. The detailed information for each sampling region can be found in Table S1.
**Figure S4:** CpDNA‐*rbc*L inferred 9 lineages (A‐I) in the red seaweeds *G. furcata* sensu lato and their distributions in the northwest Pacific. The detailed information for each population number can be found in Table S1.
**Figure S5:** Estimates of historical genetic exchange between major regions in the northwest Pacific for the red seaweeds *G. furcata* sensu lato. Numbers above/below arrows represent migration rates in the direction of the arrow. The thickness of arrow is scaled according to the values. The detailed information for each sampling region can be found in Table S1.
**Figure S6:** MtDNA *cox*1‐based ancestral distributional reconstruction of lineages in *G. furcata* sensu lato. The blue, green, and yellow circles outside of the node represent dispersal, vicariance, and extinction events, respectively. **A**: Northern Japan (HOK, THK) and Russia (KAM); **B**: Southern Japan (KAT, SKJ, KAS, SKK, KYS) and Korea (JJK, SOK, EAK); **C**: China (GZ, ZJ, SD, LN) and Korea (WEK); **D**: Canada (BCC).
**Figure S7:** The modelled current distributional patterns of habitat suitability of *G. furcata* sensu lato in the north Pacific.

## Data Availability

The molecular data that support the findings of this study are available on the repository Dryad (DOI: 10.5061/dryad.1zcrjdg6z) with reviewer sharing link: http://datadryad.org/share/LINK_NOT_FOR_PUBLICATION/3SMMqbFW3Q2gqGsK1AeVPKPU4eDooWtJiH8Lvxe8DGw. Detailed information of mtDNA *Cox*1 and cpDNA *rbc*L haplotypes, AMOVA results, and coordinates for SDMs are provided in Tables S1–S6.
