# Supplementary material for: Incorporating Phylogeography and SDM Projection to Inform Conservation Efforts of the Foundation Seaweed Gloiopeltis furcata sensu lato in the North Pacific Under Climate Change
Source: Evol Appl. 2026 Sep 23;19(9):e70323. doi: 10.1111/eva.70323 (PMC13601661; doi:10.1111/eva.70323)
Supplement: Supplementary file 1 [file EVA-19-e70323-s001.docx]

**Incorporating phylogeography and SDM projection to inform conservation efforts of the foundation seaweed *Gloiopeltis furcata* sensu lato in the North Pacific under climate change**

Supplementary Information

**I. TABLES**

**Table S1**. MtDNA-*cox*1 inferred genetic diversity indices of *Gloiopeltis furcata* sensu lato in the North Pacific.

Regions, localities and haplotypes marked in bold represent the newly collected populations and generated *cox*1 haplotypes in this study.

| No. | Region | Sampling locality | n/*N_h_* | Haplotypes | Genbank accessions | *h* | *π* (×10^-2^) |
| --- | --- | --- | --- | --- | --- | --- | --- |
| 1 | **GZ** | **Nan’ao Island, China** | 5/2 | H47,**H157** | MN650139,PZ547762 | 0.6000±0.1753 | 0.0011±0.0012 |
| 2 | **ZJ** | **Goji Island, China** | 30/3 | **H183**,**H188**,**H189** | PZ547765, PZ547770,PZ547771 | 0.4966±0.0799 | 0.0009±0.0009 |
| 3 |  | **Sijiao Island, China** | 25/4 | **H183**,**H184**,**H185**,**H186** | PZ547765-PZ547768 | 0.2300±0.1095 | 0.0006±0.0007 |
| 4 | **SD** | **Maidao Island, China** | 3/1 | H119 | MN650134 | 0 | 0 |
| 5 |  | **Dongchu Island, China** | 31/7 | H119,**H154**,**H155**,**H156**, **H179**,**H180** | MN650134,PZ547759-PZ547761, PZ547763,PZ547764 | 0.4043±0.1106 | 0.0323±0.0164 |
| 6 |  | **Daheishan Island, China** | 30/5 | H119,**H190**,**H191**,**H192**,**H193** | MN650134,PZ547772-PZ547775 | 0.4529±0.1049 | 0.0012±0.0010 |
| 7 |  | **Beichangshan Island, China** | 16/4 | H119,**H151**,**H152**,**H153** | MN650134,PZ547756-PZ547758 | 0.4417±0.1446 | 0.0009±0.0009 |
| 8 | **LN** | **Nanhaitou, China** | 7/4 | H119,H127,**H183**,**H187** | MN650134,MN650144,PZ547765, PZ547769 | 0.7143±0.1809 | 0.0643±0.0366 |
| 9 | WEK | Sochungdo, Korea | 5/2 | H70,H127 | MN650133,MN650144 | 0.4000±0.2373 | 0.0550±0.0340 |
| 10 |  | Wido, Korea | 8/3 | H70,H119,H120 | MN650133,MN650134,MN650135 | 0.6786±0.1220 | 0.0785±0.0435 |
| 11 |  | Padori, Korea | 5/2 | H70,H131 | MN650133,MW271579 | 0.6000±0.1753 | 0.0127±0.0084 |
| 12 |  | Bangpo, Korea | 5/1 | H127 | MN650144 | 0 | 0 |
| 13 |  | Jindo, Korea | 33/8 | H70,H119,H173-H178 | MN650133,MN650134,MW271608,MW271609, | 0.7841±0.0582 | 0.0781±0.0386 |
| 14 | JJK | Hansuri, Korea | 5/1 | H47 | MN650139 | 0 | 0 |
| 15 |  | Marado, Korea | 4/1 | H47 | MN650139 | 0 | 0 |
| 16 |  | Gapado, Korea | 1/1 | H48 | MN650131 | 1 | 0 |
| 17 |  | Sagye, Korea | 1/1 | H121 | MN650132 | 1 | 0 |
| 18 |  | Haye, Korea | 1/1 | H127 | MN650133 | 1 | 0 |
| 19 |  | Seogwipo, Korea | 2/2 | H142,H143 | MW271588, MW271587 | 1.0000±0.5000 | 0.1305±0.1314 |
| 20 |  | Sungsan, Korea | 7/6 | H41,H48,H116,H117,H130, H141 | MW271586 | 0.9524±0.0955 | 0.1067±0.0603 |
| 21 |  | Seongsan, Korea | 2/1 | H141 | MW271586 | 0 | 0 |
| 22 |  | Jongdal, Korea | 7/5 | H125-H129 | MW271556,MW271557,MW271558, MW271560, MW271561 | 0.8571±0.1371 | 0.0681±0.0388 |
| 23 |  | Hamduck, Korea | 2/1 | H47 | MN650139 | 0 | 0 |
| 24 |  | Yeondae, Korea | 1/1 | H144 | MW271589 | 1 | 0 |
| 25 | SOK | Wando, Korea | 32/7 | H48,H131,H168-H172 | MN650136,MN650131,MW271598,MW271598,MW271590 | 0.6512±0.0697 | 0.0196±0.0102 |
| 26 |  | Chungsando, Korea | 1/1 | H119 | MN650134 | 1 | 0 |
| 27 |  | Geomundo, Korea | 8/4 | H135-H138 | MN650149,MN650150,MN650151,MN650152 | 0.7500±0.1391 | 0.0026±0.0020 |
| 28 |  | Heungnam, Korea | 5/2 | H139,H140 | MW271585,MW271584 | 0.4000±0.2373 | 0.0007±0.0009 |
| 29 |  | Gijang, Korea | 21/9 | H70,H125,H127,H145-H150 | MN650137,MN650183,MN650184,MN650157,MN650144,MN650186,MN650187,MN650133,MN650185 | 0.8619±0.0551 | 0.0994±0.0500 |
| 30 | EAK | Pohang, Korea | 2/1 | H70 | MN650133 | 0 | 0 |
| 31 |  | Jukbyeon, Korea | 7/2 | H123,H124 | MN650140,MN650141 | 0.5714±0.1195 | 0.0020±0.0017 |
| 32 |  | Ullengdo, Korea | 1/1 | H118 | MN650176 | 1 | 0 |
| 33 |  | Dokdo, Korea | 1/1 | H47 | MN650139 | 1 | 0 |
| 34 |  | Munamjin, Korea | 3/1 | H122 | MN650132 | 0 | 0 |
| 35 |  | Hajodae, Korea | 3/3 | H132-H134 | MN650147,MN650148,MN650177 | 1.0000±0.2722 | 0.0024±0.0024 |
| 36 | HOK | Shizuura, Japan | 15/4 | H1-H4 | MW271591,MW271594,PX436326,MW271597 | 0.4725±0.1358 | 0.4667±0.1478 |
| 37 |  | Ishizaki, Japan | 14/3 | H2,H5,H6 | MW271592,MW271593,PX436326, | 0.4725±0.1358 | 0.0009±0.0009 |
| 38 |  | Udomari, Japan | 18/2 | H5,H7 | MW271592,MW271528, | 0.5294±0.0404 | 0.0719±0.0367 |
| 39 |  | Sukki, Japan | 10/1 | H8 | MW271595 | 0 | 0 |
| 40 |  | Sakaehama, Japan | 9/2 | H2,H8 | MW271595,PX436326 | 0.5000±0.1283 | 0.0018±0.0015 |
| 41 |  | Masadomari, Japan | 20/1 | H2 | PX436326 | 0 | 0 |
| 42 |  | Shikishimanai, Japan | 20/3 | H2,H8,H9 | MW271595,MW271603,PX436326 | 0.2789±0.1235 | 0.0016±0.0013 |
| 43 |  | Tomari, Japan | 3/2 | H2,H7 | MW271528,PX436326 | 0.6667±0.3143 | 0.0894±0.0674 |
| 44 |  | Oshoro, Japan | 1/1 | H2 | PX436326 | 1 | 0 |
| 45 |  | Mashike, Japan | 7/2 | H8,H98 | MW271602,MW271595 | 0.2857±0.1964 | 0.0005±0.0007 |
| 46 |  | Habomai, Japan | 12/3 | H110-H112 | MW271605,MW271606,MW271607 | 0.4394±0.1581 | 0.0055±0.0035 |
| 47 |  | Muroran, Japan | 6/2 | H13,H77 | MN650174,MN650146 | 0.5333±0.1721 | 0.0762±0.0447 |
| 48 |  | Hakodate, Japan | 16/11 | H7,H13,H70-H78 | MN650174,MW271531,MW271534,MN650175,MW271533,MW271535,MW271530,MW271528,MW271529,MN650133,MN650146 | 0.9500±0.0364 | 0.0943±0.0482 |
| 49 |  | Moheji, Japan | 9/2 | H7,H11 | MW271528,PX436322 | 0.3889±0.1644 | 0.0007±0.0008 |
| 50 |  | Nakanokawa, Japan | 1/1 | H13 | MN650146 | 1 | 0 |
| 51 |  | Shiragami, Japan | 14/4 | H7,H11,H13,H97 | MW271528,PX436322,MW271604,MN650146 | 0.5714±0.1322 | 0.0228±0.0123 |
| 52 | THK | Shiriya, Japan | 3/1 | H11 | PX436322 | 0 | 0 |
| 53 |  | Iwaya, Japan | 7/3 | H11,H108,H109 | PX436322,MW271536,MW271537 | 0.3778±0.1813 | 0.0007±0.0008 |
| 54 |  | Shimokita, Japan | 15/6 | H11,H13,H70,H81-H83 | PX436322,MW271538,MW271539,MW271540,MN650133,MN650146 | 0.8000±0.0771 | 0.0857±0.0441 |
| 55 |  | Ippongi, Japan | 15/4 | H7,H11,H99,H100 | PX436322,MW271528,MW271600,MW271601 | 0.6571±0.0800 | 0.0014±0.0012 |
| 56 |  | Imabetsu, Japan | 8/2 | H11,H104 | PX436322,MW271610 | 0.2500±0.1802 | 0.0004±0.0006 |
| 57 |  | Tappizaki, Japan | 9/4 | H7,H11,H101,H102 | PX436322,PX436330,MW271528, MW271529 | 0.7500±0.1121 | 0.0022±0.0017 |
| 58 |  | Aoiwa, Japan | 8/1 | H103 | MW271596 | 0 | 0 |
| 59 |  | Iwate, Japan | 8/5 | H10-H14 | PX436289,PX436320,PX436321,PX436322,MN650146 | 0.7857±0.1508 | 0.0690±0.0383 |
| 60 |  | Onagawa, Japan | 15/1 | H11 | PX436322 | 0 | 0 |
| 61 |  | Shichigahama, Japan | 12/1 | H11 | PX436322 | 0 | 0 |
| 62 | KAT | Katsuura, Japan, Japan | 7/4 | H13,H113-H115 | MN650146,MN650171,MN650172,MN650179 | 0.8095±0.1298 | 0.0024±0.0019 |
| 63 |  | Tateyama, Japan | 58/3 | H34,H158,H182 | MN650193,PX436340,PX436336 | 0.1010±0.0535 | 0.0097±0.0053 |
| 64 | SKJ | Shikinejima, Japan | 14/3 | H105-H107 | MW271611,MW271612,MW271613 | 0.3846±0.1494 | 0.0017±0.0014 |
| 65 | KAS | Mie, Japan | 19/8 | H54,H55,H87-H92 | PX436310,PX436311,MW271544,MW271545,MW271546,MW271547,MW271548,MW271549 | 0.8772±0.0442 | 0.0044±0.0028 |
| 66 |  | Kishu, Japan | 16/4 | H54-H57 | PX436298,PX436299,PX436310,PX436311 | 0.5167±0.1324 | 0.0015±0.0012 |
| 67 |  | Kozai, Japan | 16/2 | H58,H59 | PX436300,PX436301 | 0.1250±0.1064 | 0.0002±0.0004 |
| 68 |  | Hioki, Japan | 12/5 | H60-H64 | PX436302,PX436303,PX436304,PX436305,PX436306 | 0.8485±0.0586 | 0.0027±0.0020 |
| 69 |  | Shirahama, Japan | 18/3 | H60,H65,H66 | PX436302,PX436307,PX436308 | 0.3072±0.1316 | 0.0006±0.0007 |
| 70 | SKK | Yura, Japan | 17/2 | H61,H67 | PX436303,PX436309 | 0.1176±0.1012 | 0.0002±0.0004 |
| 71 |  | Hiwasa, Japan | 14/5 | H13,H34,H42,H79,H80 | PX436295,PX436332,MN650146,MW271536,MW271559 | 0.8352±0.0568 | 0.0964±0.0498 |
| 72 |  | Tosa, Japan | 19/4 | H42,H84-H86 | PX436295,PX436343,PX436342, PX436313 | 0.7135±0.0582 | 0.0521±0.0267 |
| 73 |  | Tsuwachijima, Japan | 15/9 | H18,H22-H29 | PX436323,PX436339,MN650171,MN650172,MN650179,MG209327,MG209328,MG209329,MG209330 | 0.8857±0.0686 | 0.0844±0.0434 |
| 74 |  | Ohshima, Japan | 13/7 | H18,H23,H26,H27,H33-H35 | PX436315,PX436323,PX436331,PX436332,PX436339,PX436325,PX436326 | 0.7949±0.1091 | 0.0637±0.0334 |
| 75 |  | Seto, Japan | 6/5 | H18,H26,H30-H32 | PX436292,PX436323,PX436327,PX436339,PX436341 | 0.9333±0.1217 | 0.1050±0.0613 |
| 76 | SKK | Misaki, Japan | 10/6 | H23,H26,H36-H39 | PX436293,PX436294,PX436315,PX436317,PX436339,PX436342 | 0.8444±0.1029 | 0.0932±0.0500 |
| 77 | KYS | Saeki, Japan | 7/5 | H17-H21 | PX436290,PX436291,PX436314,PX436323,PX436324 | 0.9048±0.1033 | 0.1003±0.0567 |
| 78 |  | Amakusa, Japan | 17/5 | H42,H93-H96 | PX436295, MW271580, MW271581, MW271582, MW271583 | 0.5074±0.1403 | 0.0320±0.0167 |
| 79 |  | Kayaki, Japan | 21/6 | H16,H42,H52-H55 | PX436295,PX436297,PX436310,PX436311,PX436337,PX436338 | 0.7476±0.0692 | 0.0848±0.0427 |
| 80 |  | Arikawa, Japan | 18/8 | H42,H45-H51 | PX436295,PX436296,MN650139,PX436319,PX436333,PX436334,PX436335,MN650136 | 0.8497±0.0547 | 0.0977±0.0496 |
| 81 |  | Tachiura, Japan | 11/6 | H16,H40-H44 | PX436329,MW271551,MW271552, MW271553,MW271554,MW271555 | 0.8000±0.1138 | 0.1054±0.0557 |
| 82 |  | Nagasaki, Japan | 4/3 | H13,H15,H16 | PX436316,PX436318, PX436328 | 0.8333±0.2224 | 0.0764±0.0506 |
| 83 |  | Kadoshima, Japan | 12/3 | H68-H70 | PX436312,MW271526,MN650133 | 0.5303±0.1359 | 0.0734±0.0386 |
| 84 | KAM | Kamchatka, Russia | 1/1 | H167 | MN650162 | 1 | 0 |
| 85 | BCC* | Canada | 31/8 | H159-H166 | MN650163, MN650164, MN650165, MN650166, MN650167, MN650168  MN650169, MN650170 | 0.6129±0.0948 | 0.0014±0.0012 |

Genetic diversity indices---n: number of sequences; *N_h_*: number of haplotypes; *h*: haplotype diversity; *π*: nucleotide diversity

Region---GZ: Guangzhou, China; ZJ: Zhejiang, China; SD: Shandong, China; LN: Liaoning, China; WEK, West coast of Korea; JJK, Jeju Island, Korea; SOK, South coast of Korea; EAK, East coast of Korea; HOK, Hokkaido, Japan; THK, Tohoku, Japan; KAT, Kanto, Japan; SKJ, Shikinejima, Japan; KAS, Kansai, Japan; SKK, Shikoku, Japan; KYS, Kyushu, Japan; KAM: Kamchatka, Russia; BCC: British Columbia, Canada

*31 samples collected from 22 distinct locations in British Columbia, Canada were pooled as a single population.

**Table S2**. CpDNA-*rbc*L inferred genetic diversity indices of *Gloiopeltis furcata* sensu lato in the North Pacific.

Regions, localities and haplotypes marked in bold represent the newly collected populations and generated *rbc*L haplotypes in this study.

| No. | Region | Sampling locality | n/*N_h_* | Haplotypes | Genbank accessions | *h* | *π* (×10^-2^) |
| --- | --- | --- | --- | --- | --- | --- | --- |
| 1 | **GZ** | **Nan’ao Island, China** | 7/2 | H1,**H2** | MN720461,PZ547776 | 0.2857±0.1964 | 0. 0005±0.0005 |
| 2 | **ZJ** | **Goji Island, China** | 29/1 | **H3** | PZ547777 | 0 | 0 |
| 3 |  | **Sijiao Island, China** | 10/3 | **H3**,**H4**,**H5** | PZ547777-PZ547779 | 0.5111±0.1643 | 0.0009±0.0070 |
| 4 | **SD** | **Maidao Island, China** | 6/2 | **H6**,H7 | MN720463,PZ547780 | 0.5333±0.1721 | 0.0004±0.0005 |
| 5 |  | **Dongchu Island, China** | 27/4 | H7,**H8**,**H9**,**H10** | MN720463,PZ547781-PZ547783 | 0.0004± 0.0005 | 0.0235±0.0118 |
| 6 |  | **Daheishan Island, China** | 30/1 | H7 | MN720463 | 0 | 0 |
| 7 |  | **Beichangshan Island, China** | 12/4 | H7,**H11**,**H12**,**H13** | MN720463,PZ547784-PZ547786 | 0.5606±0.1540 | 0.0005±0.0005 |
| 9 | WEK | Sochungdo, Korea | 5/3 | H14-H16 | MN720491,MN720471-MN720473 | 0.7000±0.2184 | 0.0201±0.0125 |
| 10 |  | Wido, Korea | 9/3 | H7,H14,H17 | MN720463 | 0.6389±0.1258 | 0.0281±0.0154 |
| 11 |  | Padori, Korea | 3/1 | H18 | MN720487 | 0 | 0 |
| 12 |  | Bangpo, Korea | 6/3 | H19-H21 | MN720461 | 0.7333±0.1552 | 0.0021±0.0015 |
| 13 |  | Jindo, Korea | 20/7 | H7,H14,H17,H22-H25 | MN720463,MN720492,MN720480,MN720460 | 0.7263±0.0917 | 0.0174±0.0089 |
| 15 | JJK | Marado, Korea | 4/1 | H1 | MN720461 | 0 | 0 |
| 17 |  | Sagye, Korea | 1/1 | H26 | MN720467 | 1 | 0 |
| 18 |  | Haye, Korea | 1/1 | H20 | MN720494 | 1 | 0 |
| 19 |  | Seogwipo, Korea | 5/5 | H27-H31 | MN720475-MN720478 | 1.0000±0.1265 | 0.0428±0.0263 |
| 20 |  | Sungsan, Korea | 1/1 | H26 | MN720467 | 1 | 0 |
| 22 |  | Jongdal, Korea | 7/3 | H21,H32-H33 | MN720474,MN720475 | 0.6667±0.1598 | 0.0614±0.0347 |
| 24 |  | Yeondae, Korea | 2/1 | H1 | MN720461 | 0 | 0 |
| 25 | SOK | Wando, Korea | 28/3 | H14,H18,H34 | MN720470 | 0.3704±0.1046 | 0.0004±0.0004 |
| 26 |  | Chungsando, Korea | 2/1 | H7 | MN720463 | 0 | 0 |
| 27 |  | Geomundo, Korea | 8/1 | H1 | MN720461 | 0 | 0 |
| 28 |  | Heungnam, Korea | 2/2 | H15,H35 | MN720487,MN720470 | 1.0000±0.5000 | 0.0016±0.0020 |
| 29 |  | Gijang, Korea | 33/7 | H14-H15,H32,H36-H39 | MN720481-MN720485 | 0.8295±0.0279 | 0.0315±0.0156 |
| 31 | EAK | Jukbyeon, Korea | 28/3 | H14,H18,H34 | MG209360 | 0.3704±0.1046 | 0.0004±0.0004 |
| 33 |  | Dokdo, Korea | 1/1 | H1 | MN720461 | 1 | 0 |
| 34 |  | Munamjin, Korea | 3/1 | H42 | MG209362 | 0 | 0 |
| 47 | HOK | Muroran, Japan | 11/5 | H14,H43-H46 | MN720496,MN720497,MN720490,MN720479 | 0.7091±0.1366 | 0.0328±0.0174 |
| 62 | KAT | Katsuura, Japan | 6/2 | H43,H47 | MN720495,MN720486 | 0.3333±0.2152 | 0.0003±0.0004 |
| 63 |  | Tateyama, Japan | 50/2 | H43,H48 | MG209363,MG209364 | 0.0784±0.0510 | 0.0001±0.0001 |
| 85 | BCC | Canada | 1/1 | H49 | MN720498 | 1 | 0 |

Genetic diversity indices---n: number of sequences; *N_h_*: number of haplotypes; *h*: haplotype diversity; *π*: nucleotide diversity

Region---GZ: Guangzhou, China; ZJ: Zhejiang, China; SD: Shandong, China; WEK, West coast of Korea; JJK, Jeju Island, Korea; SOK, South coast of Korea; EAK, East coast of Korea; HOK, Hokkaido, Japan; KAT, Kanto, Japan; BCC: British Columbia, Canada

**Table S3**. MtDNA-*cox*1 based Analysis of Molecular variance (AMOVA) for *G. furcata* sensu lato in the north Pacific using multiple grouping criteria.

| Grouping criteria | Among groups | | | Among populations within groups | | | | Within populations | | | |
| --- | --- | --- | --- | --- | --- | --- | --- | --- | --- | --- | --- |
|  | d.f. | Var(%) | F_CT_ | d.f. | Var(%) | F_SC_ | d.f. | | | Var(%) | F_ST_ |
| Country | 4 | 24.80% | 0.2480*** | 80 | 50.63% | 0.6733*** | 889 | | 24.57% | | 0.7543*** |
| Longitude-1 | 1 | 11.30% | 0.1130*** | 83 | 62.57% | 0.7054*** | | 889 | | 26.14% | 0.7387** |
| Longitude-2 | 1 | 19.68% | 0.1968*** | 83 | 57.25% | 0.7128*** | | 889 | | 23.07% | 0.7693*** |
| Marine Ecoregions-1 | 1 | 14.51% | 0.1451*** | 82 | 59.07% | 0.6909*** | | 851 | | 26.43% | 0.7358** |
| Marine Ecoregions-2 | 2 | 13.18% | 0.1318*** | 82 | 60.65% | 0.6985*** | | 881 | | 26.17% | 0.7383** |
| Marine Provinces-1 | 1 | 17.96% | 0.1796*** | 82 | 57.00% | 0.6948*** | | 859 | | 25.04% | 0.7496** |
| Marine Provinces-2 | 1 | 18.83% | 0.1883*** | 83 | 56.84% | 0.7003*** | | 889 | | 24.32% | 0.7568*** |
| Biogeographic basins-1 | 3 | 25.90% | 0.2590*** | 81 | 48.49% | 0.6543*** | | 889 | | 25.62% | 0.7438*** |
| Biogeographic basins-2 | 2 | 20.96% | 0.2096*** | 82 | 53.35% | 0.6750*** | | 889 | | 25.69% | 0.7431*** |
| Biogeographic basins-3 | 4 | 25.45% | 0.2545*** | 80 | 48.71% | 0.6534*** | | 889 | | 25.84% | 0.7416*** |

d.f.: degree of freedom; Var(%): percentage of variation; F: fixation index

**: *P* < 0.001; ***: *P* < 0.0001

**Country**: group1--China, populations 1-8; group2--Korea, populations 9-35; group3--Japan, populations 36-83; group4--Russia, population 84; group5--Canada, population 85

**Longitude-1**: group1--35°N south, populations 1-3, 13-28, 63-83; group2--35°N north, populations 4-12, 29-62, 84-85

**Longitude-2**: group1--40°N south, populations 1-35, 59-83; group2--40°N north, populations 36-58, 84-85

**Marine Ecoregions-1**: group1--Cold Temperate Northwest Pacific, populations 4-8, 9-13, 30-61, 84; group2--Warm Temperate Northwest Pacific, populations 2-3, 14-29, 62-83

**Marine Ecoregions-2**: group1--Cold Temperate Northwest Pacific, populations 4-8, 9-13, 30-61, 84; group2--Warm Temperate Northwest Pacific, populations 2-3, 14-29, 62-83; group3--Warm Temperate Northeast Pacific, population 85

**Marine provinces-1**: group1--Kuroshio basin, populations 14-29, 36-83; group2--Coastal currents, populations 1-13, 30-35, 84

**Marine provinces-2**: group1--warm current, populations 14-29, 36-83, 85; group2--cold current, populations 1-13, 30-35, 84

**Biogeographic basins-1**: group1--China Sea, populations 1-13; group2--Tsushima Strait, populations 14-29, 80-83; group3--Sea of Japan, populations 30-45, 49, 51, 55-58; group4--Pacific Rim, populations 46-48, 50, 52-54, 59-79, 84-85

**Biogeographic basins-2**: group1--China Sea, populations 1-29, 78-82; group2--Sea of Japan, populations 30-45, 49, 51, 55-58, 83; group3--Pacific Rim, populations 46-48, 50, 52-54, 59-77, 84-85

**Biogeographic basins-3**: group1--China Sea, populations 1-13; group2--Tsushima Strait, populations 14-29, 80-83; group3--Sea of Japan, populations 30-45, 49, 51, 55-58; group4--west bank of Pacific Ocean, populations 46-48, 50, 52-54, 59-79, 84; group5--east bank of Pacific Ocean, populations 85

**Table S4**. CpDNA-*rbc*L based Analysis of Molecular variance (AMOVA) for *G. furcata* sensu lato in the north Pacific using multiple grouping criteria.

| Grouping criteria | Among groups | | | Among populations within groups | | | | Within populations | | | |
| --- | --- | --- | --- | --- | --- | --- | --- | --- | --- | --- | --- |
|  | d.f. | Var(%) | F_CT_ | d.f. | Var(%) | F_SC_ | d.f. | | | Var(%) | F_ST_ |
| Country | 3 | 8.87% | 0.0887*** | 28 | 71.05% | 0.7797*** | 304 | | 20.08% | | 0.7992*** |
| Longitude-1 | 1 | -9.1% | -0.0910^ns^ | 30 | 87.58% | 0.8028*** | | 304 | | 21.52% | 0.7848*** |
| Marine Ecoregions-1 | 1 | 3.56% | 0.0356*** | 28 | 75.89% | 0.7869*** | | 298 | | 20.55% | 0.7945*** |
| Marine Ecoregions-2 | 2 | 3.86% | 0.0386*** | 28 | 75.65% | 0.7869*** | | 298 | | 20.49% | 0.7951*** |
| Marine Provinces-1 | 1 | 5.01% | 0.0501*** | 29 | 74.78% | 0.7873*** | | 304 | | 20.21% | 0.7979*** |
| Marine Provinces~2 | 1 | 4.91% | 0.0491*** | 30 | 74.91% | 0.7878*** | | 304 | | 20.18% | 0.7982*** |
| Biogeographic basins-1 | 2 | 10.55% | 0.1056*** | 29 | 70.11% | 0.7839*** | | 304 | | 19.33% | 0.8067*** |
| Biogeographic basins-2 | 3 | 3.27% | 0.0327*** | 28 | 76.29% | 0.7887*** | | 304 | | 20.44% | 0.7956*** |
| Biogeographic basins-3 | 4 | 3.53% | 0.0353*** | 27 | 76.04% | 0.7883*** | | 304 | | 20.42% | 0.7958*** |

d.f.: degree of freedom; Var(%): percentage of variation; F: fixation index

***: *P* < 0.0001; ns: non-significant

**Country**: group1--China, populations 1-8; group2--Korea, populations 9-35; group3--Japan, populations 36-83; group4--Russia, population 84; group5--Canada, population 85

**Longitude-1**: group1--35°N south, populations 1-3, 13-28, 63-83; group2--35°N north, populations 4-12, 29-62, 84-85

**Marine Ecoregions-1**: group1--Cold Temperate Northwest Pacific, populations 4-8, 9-13, 30-61, 84; group2--Warm Temperate Northwest Pacific, populations 2-3, 14-29, 62-83

**Marine Ecoregions-2**: group1--Cold Temperate Northwest Pacific, populations 4-8, 9-13, 30-61, 84; group2--Warm Temperate Northwest Pacific, populations 2-3, 14-29, 62-83; group3--Warm Temperate Northeast Pacific, population 85

**Marine provinces-1**: group1--Kuroshio basin, populations 14-29, 36-83; group2--Coastal currents, populations 1-13, 30-35, 84

**Marine provinces-2**: group1--warm current, populations 14-29, 36-83, 85; group2--cold current, populations 1-13, 30-35, 84

**Biogeographic basins-1**: group1--China Sea, populations 1-13; group2--Tsushima Strait, populations 14-29, 80-83; group3--Sea of Japan, populations 30-45, 49, 51, 55-58; group4--Pacific Rim, populations 46-48, 50, 52-54, 59-79, 84-85

**Biogeographic basins-2**: group1--China Sea, populations 1-29, 78-82; group2--Sea of Japan, populations 30-45, 49, 51, 55-58, 83; group3--Pacific Rim, populations 46-48, 50, 52-54, 59-77, 84-85

**Biogeographic basins-3**: group1--China Sea, populations 1-13; group2--Tsushima Strait, populations 14-29, 80-83; group3--Sea of Japan, populations 30-45, 49, 51, 55-58; group4--west bank of Pacific Ocean, populations 46-48, 50, 52-54, 59-79, 84; group5--east bank of Pacific Ocean, populations 85

**Table S5**. Occurrence records of the red seaweeds *G. furcata* sensu lato in the north Pacific from literatures used for species distribution modelling (SDM).

| No. (Sites) | Locality | Country | Latitude | Longitude |
| --- | --- | --- | --- | --- |
| 1 | Shizuura | Japan | 41°29'28"N | 140°00'55"E |
| 2 | Ishizaki | Japan | 41°48'22"N | 140°04'24"E |
| 3 | Udomari | Japan | 42°21'27"N | 139°47'46"E |
| 4 | Sukki | Japan | 42°35'41"N | 139°49'31"E |
| 5 | Sakaehama | Japan | 42°40'11"N | 139°52'47"E |
| 6 | Masadomari | Japan | 42°48'42"N | 140°10'40"E |
| 7 | Shikishimanai | Japan | 42°57'43"N | 140°27'45"E |
| 8 | Tomari | Japan | 43°04'03"N | 140°29'29"E |
| 9 | Oshoro | Japan | 43°12'20"N | 140°52'23"E |
| 10 | Mashike | Japan | 43°51'09"N | 141°32'11"E |
| 11 | Habomai | Japan | 43°20'55"N | 145°46'25"E |
| 12 | Moheji | Japan | 41°45'38"N | 140°36'15"E |
| 13 | Shiragami | Japan | 41°23'53"N | 140°11'57"E |
| 14 | Muroran | Japan | 42°20'00.8"N | 141°01'33.1"E |
| 15 | Hakodate | Japan | 41°43'09"N | 141°01'30"E |
| 16 | Nakanokawa | Japan | 41°38'08"N | 140°25'54"E |
| 17 | Shiriya | Japan | 41°25'49"N | 141°27'45"E |
| 18 | Iwaya | Japan | 41°23'37"N | 141°24'55"E |
| 19 | Shimokita | Japan | 41°27'37"N | 141°06'41"E |
| 20 | Ippongi | Japan | 41°13'07"N | 140°32'24"E |
| 21 | Imabetsu | Japan | 41°11'02"N | 140°29'06"E |
| 22 | Tappizaki | Japan | 41°15'11"N | 140°20'28"E |
| 23 | Aoiwa | Japan | 41°09'49"N | 140°19'39"E |
| 24 | Iwate | Japan | 39°25'29"N | 141°58'52"E |
| 25 | Shichigahama | Japan | 38°16'58"N | 141°03'48"E |
| 26 | Onagawa | Japan | 38°28'13"N | 141°29'01"E |
| 27 | Katssura | Japan | 35°09'00.5"N | 140°18'23.6"E |
| 28 | Tateyama | Japan | 34°59'26.9"N | 139°49'36.3"E |
| 29 | Shikinejima | Japan | 34°19'56"N | 139°12'52"E |
| 30 | Mie | Japan | 33°53'23"N | 136°06'34"E |
| 31 | Kishu | Japan | 33°37'57"N | 135°56'53"E |
| 32 | Kozai | Japan | 33°30'49"N | 135°49'46"E |
| 33 | Hioki | Japan | 33°33'25"N | 135°26'34"E |
| 34 | Shirahama | Japan | 33°41'01"N | 135°20'32"E |
| 35 | Yura | Japan | 33°57'37"N | 135°06'51"E |
| 36 | Hiwasa | Japan | 33°43'54"N | 134°32'29"E |
| 37 | Tosa | Japan | 33°27'15"N | 133°28'09"E |
| 38 | Tsuwachijima | Japan | 33°59'00"N | 132°31'07"E |
| 39 | Ohshima | Japan | 33°23'37"N | 132°20'42"E |
| 40 | Seto | Japan | 33°25'30"N | 132°13'16"E |
| 41 | Misaki | Japan | 33°23'27"N | 132°06'54"E |
| 42 | Amakusa | Japan | 32°22'58"N | 129°59'38"E |
| 43 | Kayaki | Japan | 32°39'21"N | 129°48'57"E |
| 44 | Arikawa | Japan | 32°59'00"N | 129°06'28"E |
| 45 | Tachiura | Japan | 33°21'22"N | 129°26'02"E |
| 46 | Kadoshima | Japan | 34°21'12"N | 130°54'02"E |
| 47 | Saeki | Japan | 32°58'48"N | 131°54'15"E |
| 48 | Nagasaki | Japan | 33°45'02"N | 129°47'16"E |
| 49 | Sochungdo | Korea | 37°45'51.7"N | 124°45'34.8"E |
| 50 | Wido | Korea | 35°36'13.8"N | 126°16'56.1"E |
| 51 | Padori | Korea | 36°44'20.6"N | 126°07'56.8"E |
| 52 | Bangpo | Korea | 36°30'35.3"N | 126°19'56.0"E |
| 53 | Jindo | Korea | 34°23'05.5"N | 126°17'47.9"E |
| 54 | Hansuri | Korea | 33°25'26.7"N | 126°15'37.5"E |
| 55 | Seogwipo | Korea | 33°13'37.9"N | 126°33'50.3"E |
| 56 | Sungsan | Korea | 33°27'09.1"N | 126°55'32.6"E |
| 57 | Sagye | Korea | 33°13'52.4"N | 126°18'38.8"E |
| 58 | Jongdal | Korea | 33°29'27.5"N | 126°54'43.6"E |
| 59 | Yeondae | Korea | 33°29'43.0"N | 126°25'33.2"E |
| 60 | Hamduck | Korea | 33°32'45.8"N | 126°40'25.7"E |
| 61 | Haye | Korea | 33°13'58.7"N | 126°22'25.6"E |
| 62 | Gapado | Korea | 33°10'30.9"N | 126°16'19.9"E |
| 63 | Marado | Korea | 33°07'24.0"N | 126°16'10.1"E |
| 64 | Seongsan | Korea | 53°27'44"N | 126°56'18"E |
| 65 | Wando | Korea | 34°17'50.8"N | 126°42'10.6"E |
| 66 | Chungsando | Korea | 34°11'54.3"N | 126°51'40.9"E |
| 67 | Geomundo | Korea | 34°03'20.3"N | 127°17'42.1"E |
| 68 | Gijang | Korea | 35°15'46.5"N | 129°14'03.2"E |
| 69 | Heungnam | Korea | 34°57'34.6"N | 128°42'54.9"E |
| 70 | Pohang | Korea | 35°59'49.5"N | 129°33'59.9"E |
| 71 | Jukbyeon | Korea | 37°03'28.4"N | 129°25'48.7"E |
| 72 | Ullengdo | Korea | 37°30'27.0"N | 130°54'50.4"E |
| 73 | Dokdo | Korea | 37°14'21.8"N | 131°52'04.9"E |
| 74 | Munamjin | Korea | 38°17'48.1"N | 128°32'59.9"E |
| 75 | Hajodae | Korea | 38°01'21.3"N | 128°43'43.0"E |
| 76 | Nanhaitou, Dalian | China | 39°21'52.5"N | 121°24'1.8"E |
| 77 | Dongchu Island, Rongcheng | China | 37°23'30"N | 123°38'39"E |
| 78 | Maidao Island, Qingdao | China | 36°03'16.6"N | 120°25'41.0"E |
| 79 | Daheishan Mountain, Yantai | China | 37°58'39.7"N | 120°37'38.0"E |
| 80 | Beichangshan Island, Yantai | China | 37°59'20"N | 120°42'17"E |
| 81 | Sijiao Island, Zhoushan | China | 30°43'8.1"N | 122°30'41.6"E |
| 82 | Goji Island, Zhoushan | China | 30°42'51.8"N | 122°46'17.1"E |
| 83 | Nan’ao Island, Shantou | China | 23°26'04.4"N | 117°07'58.7"E |
| 84 | Kamchatka | Russia | 52°46'47"N | 158°36'50"E |
| 85 | Seppings Is., Bamfield | Canada | 48°50'20.8"N | 125°12'27.0"W |
| 86 | Dixon Is., Bamfield | Canada | 48°51'08.6"N | 125°07'20.6"W |
| 87 | Prince Rupert, Seal Cove | Canada | 54°19'57.0"N | 130°17'00.2"W |
| 88 | Prince Rupert, Ridley Is. | Canada | 54°13'16.3"N | 130°19'45.5"W |
| 89 | Willow Point, Campbell River | Canada | 49°57'58.3"N | 125°12'29.2"W |
| 90 | Friendly Cove, Tahsis | Canada | 49°35'35.8"N | 126°36'59.4"W |
| 91 | Pachena Bay, Bamfield | Canada | 48°46'17.4"N | 125°09'28.1"W |
| 92 | Bradys beach, Bamfield | Canada | 48°49'26.4"N | 125°09'43.6"W |
| 93 | Halfway beach | Canada | 49°36'13.2"N | 123°49'37.8"W |
| 94 | Esperenza Inlet: Tahsis | Canada | 49°48'45.0"N | 126°59'14.4"W |
| 95 | Queen Charlotte City, Haida Gwaii | Canada | 53°14'58.5"N | 132°07'00.3"W |
| 96 | Burnaby Is., Gwaii Haanas | Canada | 52°26'57.7"N | 131°16'59.3"W |
| 97 | Huxley Is., Gwaii Haanas | Canada | 52°25'59.7"N | 131°22'13.4"W |
| 98 | Lawn Point, Haida Gwaii | Canada | 53°24'58.8"N | 131°54'56.9"W |
| 99 | Koga Islet, Gwaii Haanas | Canada | 52°25'41.9"N | 131°22'43.0"W |
| 100 | Hot Spring Is., Gwaii Haanas | Canada | 52°34'40.4"N | 131°26'15.7"W |
| 101 | Skidegate Boat Launch, Haida Gwaii | Canada | 53°15'02.1"N | 132°02'15.7"W |
| 102 | Masset Inlet, Haida Gwaii | Canada | 53°40'27.6"N | 132°22'37.5"W |
| 103 | Alder Island, Gwaii Haanas | Canada | 52°26'31.6"N | 131°19'07.9"W |
| 104 | Murchison Is., Gwaii Haanas | Canada | 52°36'21.1"N | 131°25'58.4"W |
| 105 | Woodruff Bay, Gwaii Haanas | Canada | 51°58'26.9"N | 131°01'56.0"W |
| 106 | Westacott Pt., Haida Gwaii | Canada | 54°04'40.3"N | 132°14'39.5"W |

**Table S6**. Distribution of mtDNA-*cox*1 inferred lineages (L1-L11) of *G. furcata* sensu lato in each population. Numbers in shaded indicate haplotypes in a specific lineage either distributed proximately (in bold) or endemic in 1-2 populations (in red).

| Region | Population | L1 | L2 | L3 | L4 | L5 | L6 | L7 | L8 | L9 | L10 | L11 |
| --- | --- | --- | --- | --- | --- | --- | --- | --- | --- | --- | --- | --- |
| GZ | Nan’ao Island | 3 | 0 | 0 | 2 | 0 | 0 | 0 | 0 | 0 | 0 | 0 |
| ZJ | Goji Island | 0 | 0 | 0 | 30 | 0 | 0 | 0 | 0 | 0 | 0 | 0 |
|  | Sijiao Island | 0 | 0 | 0 | 25 | 0 | 0 | 0 | 0 | 0 | 0 | 0 |
| SD | Maidao Island | 0 | 0 | 0 | 0 | 0 | 0 | 0 | 0 | 0 | 0 | **3** |
|  | Dongchu Island | 0 | 0 | 0 | 3 | 0 | 0 | 0 | 0 | 4 | 0 | **24** |
|  | Daheishan Island | 0 | 0 | 0 | 8 | 0 | 0 | 0 | 0 | 0 | 0 | **22** |
|  | Beichangshan Island | 0 | 0 | 0 | 4 | 0 | 0 | 0 | 0 | 0 | 0 | **12** |
| LN | Nanhaitou | 0 | 0 | 0 | 1 | 0 | 1 | 0 | 0 | 4 | 0 | **1** |
| WEK | Sochungdo | 4 | 0 | 0 | 0 | 0 | 0 | 0 | 0 | 1 | 0 | **0** |
|  | Wido | 3 | 0 | 0 | 0 | 0 | 0 | 7 | 0 | 0 | 0 | **4** |
|  | Padori | 2 | 0 | 0 | 0 | 0 | 0 | 0 | 0 | 0 | 0 | **11** |
|  | Bangpo | 0 | 0 | 0 | 0 | 0 | 0 | 0 | 0 | 5 | 0 | **0** |
|  | Jindo | 3 | 0 | 0 | 1 | 0 | 15 | 0 | 0 | 0 | 0 | **14** |
| JJK | Hansuri | 5 | 0 | 0 | 0 | 0 | 0 | 0 | 0 | 0 | 0 | 0 |
|  | Marado | 4 | 0 | 0 | 0 | 0 | 0 | 0 | 0 | 0 | 0 | 0 |
|  | Gapado | 0 | 0 | 0 | 0 | 0 | 0 | 0 | 1 | 0 | 0 | 0 |
|  | Sagye | 0 | 0 | 0 | 0 | 0 | 0 | 1 | 0 | 0 | 0 | 0 |
|  | Haye | 0 | 0 | 0 | 0 | 0 | 0 | 0 | 0 | 1 | 0 | 0 |
|  | Seogwipo | 0 | 0 | 0 | 2 | 0 | 0 | 0 | 0 | 0 | 0 | 0 |
|  | Gapado | 0 | 0 | 0 | 0 | 0 | 0 | 0 | 1 | 0 | 0 | 0 |
|  | Sagye | 0 | 0 | 0 | 0 | 0 | 0 | 1 | 0 | 0 | 0 | 0 |
|  | Haye | 0 | 0 | 0 | 0 | 0 | 0 | 0 | 0 | 1 | 0 | 0 |
|  | Seogwipo | 0 | 0 | 0 | 2 | 0 | 0 | 0 | 0 | 0 | 0 | 0 |
|  | Sungsan | 0 | 0 | 0 | 1 | 3 | 0 | 0 | 1 | 0 | 0 | 2 |
|  | Seongsan | 0 | 0 | 0 | 2 | 0 | 0 | 0 | 0 | 0 | 0 | 0 |
|  | Jongdal | 1 | 0 | 0 | 1 | 0 | 0 | 0 | 0 | 3 | 0 | 2 |
|  | Hamduck | 2 | 0 | 0 | 0 | 0 | 0 | 0 | 0 | 0 | 0 | 0 |
|  | Yeondae | 0 | 0 | 0 | 0 | 0 | 1 | 0 | 0 | 0 | 0 | 0 |
| SOK | Wando | 0 | 0 | 0 | 0 | 0 | 0 | 0 | 1 | 22 | 0 | 9 |
|  | Chungsando | 0 | 0 | 0 | 0 | 0 | 0 | 0 | 0 | 0 | 0 | 1 |
|  | Geomundo | 0 | 0 | 0 | 4 | 0 | 0 | 0 | 0 | 1 | 0 | 3 |
|  | Heungnam | 0 | 0 | 0 | 1 | 0 | 0 | 0 | 0 | 0 | 0 | 4 |
|  | Gijang | 7 | 0 | 0 | 6 | 0 | 3 | 0 | 0 | 2 | 0 | 3 |
| EAK | Pohang | 2 | 0 | 0 | 0 | 0 | 0 | 0 | 0 | 0 | 0 | 0 |
|  | Jukbyeon | 0 | 0 | 0 | 0 | 7 | 0 | 0 | 0 | 0 | 0 | 0 |
|  | Ullengdo | 0 | 0 | 0 | 0 | 0 | 0 | 0 | 0 | 0 | 0 | 1 |
|  | Dokdo | 1 | 0 | 0 | 0 | 0 | 0 | 0 | 0 | 0 | 0 | 0 |
|  | Munamjin | 0 | 0 | 0 | 0 | 0 | 0 | 3 | 0 | 0 | 0 | 0 |
|  | Hajodae | 0 | 0 | 0 | 1 | 0 | 2 | 0 | 0 | 0 | 0 | 0 |
| HOK | Shizuura | 11 | 0 | 0 | 0 | 0 | 0 | 0 | 0 | 0 | 0 | **4** |
|  | Ishizaki | 0 | 0 | 0 | 0 | 0 | 0 | 0 | 0 | 0 | 0 | **14** |
|  | Udomari | 0 | 0 | 0 | 0 | 9 | 0 | 0 | 0 | 0 | 0 | **9** |
|  | Sukki | 0 | 0 | 0 | 0 | 0 | 0 | 0 | 0 | 0 | 0 | **10** |
|  | Sakaehama | 0 | 0 | 0 | 0 | 0 | 0 | 0 | 0 | 0 | 0 | **9** |
|  | Masadomari | 0 | 0 | 0 | 0 | 0 | 0 | 0 | 0 | 0 | 0 | **20** |
|  | Shikishimanai | 0 | 0 | 0 | 0 | 0 | 0 | 0 | 0 | 0 | 0 | **20** |
|  | Tomari | 0 | 0 | 0 | 0 | 1 | 0 | 0 | 0 | 0 | 0 | **2** |
|  | Oshoro | 0 | 0 | 0 | 0 | 0 | 0 | 0 | 0 | 0 | 0 | **1** |
|  | Mashike | 1 | 0 | 0 | 0 | 0 | 0 | 0 | 0 | 0 | 0 | **6** |
|  | Habomai | 0 | 0 | **1** | 0 | 9 | 0 | 0 | 0 | 0 | 0 | **11** |
|  | Muroran | 0 | 0 | 0 | 4 | 0 | 0 | 0 | 0 | 2 | 0 | 0 |
|  | Nakanokawa | 0 | 0 | 0 | 1 | 0 | 0 | 0 | 0 | 0 | 0 | 0 |
|  | Hakodate | 10 | 0 | 0 | 1 | 0 | **1** | 0 | 0 | 2 | 0 | 2 |
|  | Moheji | 0 | 0 | 0 | 0 | 2 | **7** | 0 | 0 | 0 | 0 | 0 |
|  | Shiragami | 1 | 0 | 0 | 1 | 3 | **9** | 0 | 0 | 0 | 0 | 0 |
| THK | Shiriya | 0 | 0 | 0 | 0 | 0 | **3** | 0 | 0 | 0 | 0 | 0 |
|  | Iwaya | 0 | 0 | 0 | 0 | 2 | **8** | 0 | 0 | 0 | 0 | 0 |
|  | Shimokita | 3 | 0 | 0 | 3 | 0 | **6** | 0 | 0 | 0 | 0 | 3 |
|  | Ippongi | 1 | 0 | 0 | 0 | 7 | **7** | 0 | 0 | 0 | 0 | 0 |
|  | Hakodate | 10 | 0 | 0 | 1 | 0 | **1** | 0 | 0 | 2 | 0 | 2 |
|  | Moheji | 0 | 0 | 0 | 0 | 2 | **7** | 0 | 0 | 0 | 0 | 0 |
|  | Shiragami | 1 | 0 | 0 | 1 | 3 | **9** | 0 | 0 | 0 | 0 | 0 |
|  | Shiriya | 0 | 0 | 0 | 0 | 0 | **3** | 0 | 0 | 0 | 0 | 0 |
|  | Iwaya | 0 | 0 | 0 | 0 | 2 | **8** | 0 | 0 | 0 | 0 | 0 |
|  | Shimokita | 3 | 0 | 0 | 3 | 0 | **6** | 0 | 0 | 0 | 0 | 3 |
|  | Ippongi | 1 | 0 | 0 | 0 | 7 | **7** | 0 | 0 | 0 | 0 | 0 |
|  | Imabetsu | 0 | 0 | 0 | 0 | 7 | **1** | 0 | 0 | 0 | 0 | 0 |
|  | Tappizaki | 1 | 0 | 0 | 1 | 3 | **4** | 0 | 0 | 0 | 0 | 0 |
|  | Aoiwa | 8 | 0 | 0 | 0 | 0 | **0** | 0 | 0 | 0 | 0 | 0 |
|  | Iwate | 0 | 0 | 0 | 2 | 0 | **5** | 0 | 0 | 1 | 0 | 0 |
|  | Onagawa | 0 | 0 | 0 | 0 | 0 | **5** | 0 | 0 | 0 | 0 | 0 |
|  | Shichigahama | 0 | 0 | 0 | 0 | 0 | **12** | 0 | 0 | 0 | 0 | 0 |
| KAT | Katsuura | 0 | 0 | **3** | 3 | 1 | 0 | 0 | 0 | 0 | 0 | 0 |
|  | Tateyama | 0 | 0 | 0 | 0 | 0 | 0 | 58 | 0 | 0 | 0 | 0 |
| SKJ | Shikinejima | 0 | 0 | 0 | 0 | 2 | 0 | 0 | 0 | 0 | 0 | 11 |
| KAS | Mie | 2 | 0 | 0 | 3 | 9 | 0 | 0 | 4 | 0 | **1** | 0 |
|  | Kishu | 0 | 0 | 0 | 2 | 14 | 0 | 0 | 0 | 0 | 0 | 0 |
|  | Kozai | 1 | 0 | 0 | 0 | 15 | 0 | 0 | 0 | 0 | 0 | 0 |
|  | Hioki | 9 | 0 | 0 | 0 | 0 | 0 | 0 | 0 | 3 | 0 | 0 |
|  | Shirahama | 3 | 0 | 0 | 0 | 0 | 0 | 0 | 0 | 15 | 0 | 0 |
| SKK | Yura | 17 | 0 | 0 | 0 | 0 | 0 | 0 | 0 | 0 | 0 | 0 |
|  | Hiwasa | 0 | 0 | 0 | 6 | **2** | 0 | 2 | 0 | 0 | 0 | 4 |
|  | Tosa | 0 | 0 | 0 | 0 | **6** | 0 | 1 | 0 | 0 | 0 | 12 |
|  | Tsuwachijima | 2 | 0 | 0 | 1 | **12** | 0 | 0 | 0 | 0 | 0 | 0 |
|  | Ohshima | 1 | 0 | 0 | 0 | **11** | 0 | 7 | 0 | 0 | 0 | 0 |
|  | Seto | 0 | 0 | 0 | 1 | **5** | 0 | 0 | 0 | 0 | 0 | 0 |
|  | Misaki | 0 | 0 | 0 | 1 | **4** | 0 | 5 | 0 | 0 | 0 | 0 |
| KYS | Saeki | 1 | 0 | 0 | 1 | **4** | 0 | 1 | 0 | 0 | 0 | 0 |
|  | Amakusa | 5 | 0 | 0 | 0 | **12** | 0 | 0 | 0 | 0 | 0 | 0 |
|  | Kayaki | 0 | **1** | 0 | 1 | **10** | 9 | 0 | 0 | 0 | 0 | 0 |
|  | Arikawa | 7 | 0 | 0 | 5 | **1** | 0 | 1 | 4 | 0 | 0 | 0 |
|  | Tachiura | 1 | 0 | 0 | 1 | **7** | 2 | 0 | 0 | 0 | 0 | 0 |
|  | Nagasaki | 0 | 0 | 0 | 1 | 0 | 1 | 0 | 0 | 2 | 0 | 0 |
|  | Kadoshima | 12 | 0 | 0 | 0 | 0 | 0 | 0 | 0 | 0 | 0 | 0 |
| KAM | Kamchatka | 0 | 0 | 0 | 0 | 0 | 0 | 0 | 0 | 0 | 0 | 1 |
| BCC | Canada | 0 | 0 | 0 | 0 | 0 | 0 | 0 | 0 | 0 | 0 | 31 |

Region---GZ: Guangzhou, China; ZJ: Zhejiang, China; SD: Shandong, China; LN: Liaoning, China; WEK, West coast of Korea; JJK, Jeju Island, Korea; SOK, South coast of Korea; EAK, East coast of Korea; HOK, Hokkaido, Japan; THK, Tohoku, Japan; KAT, Kanto, Japan; SKJ, Shikinejima, Japan; KAS, Kansai, Japan; SKK, Shikoku, Japan; KYS, Kyushu, Japan; KAM: Kamchatka, Russia; BCC: British Columbia, Canada

**II. FIGURES**

**Figure S1**. 338 filtered occurrence records of the red macroalga *G. furcata* sensu lato in the north Pacific (we only consider regions shallower than 10 m).

**Figure S2**. Correlation levels between marine predictors. We computed the pairwise Pearson’s correlation coefficient (*r*) between predictors. Predictors with |*r*| > 0.7 are deemed high correlated.

SST: sea surface temperature; CV: current velocity; SSS: sea surface salinity; DO: dissolved oxygen; Bd: seawater depth; max: annual maximum value; mean: annual mean value; min: annual minimum value; range: annual range.

**Figure S3**. Parsimony median-joining network in the red seaweeds *G. furcata* sensu lato based on cpDNA-*rbc*L haplotypes, which identified 9 lineages (A-I). Circle size is proportional to population size, and each line between haplotypes represents one mutation step. The detailed information for each sampling region can be found in Table S1.

**Figure S4**. CpDNA-*rbc*L inferred 9 lineages (A-I) in the red seaweeds *G. furcata* sensu lato and their distributions in the northwest Pacific. The detailed information for each population number can be found in Table S1.

**Figure S5**. Estimates of historical genetic exchange between major regions in the northwest Pacific for the red seaweeds *G. furcata* sensu lato. Numbers above/below arrows represent migration rates in the direction of the arrow. The thickness of arrow is scaled according to the values. The detailed information for each sampling region can be found in Table S1.

**Figure S6**. MtDNA *cox*1-based ancestral distributional reconstruction of lineages in *G. furcata* sensu lato. The blue, green, and yellow circles outside of the node represent dispersal, vicariance, and extinction events, respectively.

**A**: Northern Japan (HOK, THK) and Russia (KAM); **B**: Southern Japan (KAT, SKJ, KAS, SKK, KYS) and Korea (JJK, SOK, EAK); **C**: China (GZ, ZJ, SD, LN) and Korea (WEK); **D**: Canada (BCC)

**Figure S7**. The modelled current distributional patterns of habitat suitability of *G. furcata* sensu lato in the north Pacific.
